# Machine learning to support visual auditing of home-based lateral flow immunoassay self-test results for SARS-CoV-2 antibodies

**DOI:** 10.1038/s43856-022-00146-z

**Published:** 2022-07-06

**Authors:** Nathan C. K. Wong, Sepehr Meshkinfamfard, Valérian Turbé, Matthew Whitaker, Maya Moshe, Alessia Bardanzellu, Tianhong Dai, Eduardo Pignatelli, Wendy Barclay, Ara Darzi, Paul Elliott, Helen Ward, Reiko J. Tanaka, Graham S. Cooke, Rachel A. McKendry, Christina J. Atchison, Anil A. Bharath

**Affiliations:** 1 Department of Bioengineering, Imperial College London, London, UK; 2 London Centre for Nanotechnology, University College London, London, UK; 3 School of Public Health, Imperial College London, London, UK; 4 Department of Infectious Disease, Imperial College London, London, UK; 5 Imperial College Healthcare NHS Trust, London, UK; 6 National Institute for Health Research Imperial Biomedical Research Centre, London, UK; 7 Institute of Global Health Innovation, Imperial College London, London, UK; 8 MRC Centre for Environment and Health, School of Public Health, Imperial College London, London, UK; 9 Division of Medicine, University College London, London, UK

**Keywords:** Databases, Public health

## Abstract

**Background:**

Lateral flow immunoassays (LFIAs) are being used worldwide for COVID-19 mass testing and antibody prevalence studies. Relatively simple to use and low cost, these tests can be self-administered at home, but rely on subjective interpretation of a test line by eye, risking false positives and false negatives. Here, we report on the development of ALFA (Automated Lateral Flow Analysis) to improve reported sensitivity and specificity.

**Methods:**

Our computational pipeline uses machine learning, computer vision techniques and signal processing algorithms to analyse images of the Fortress LFIA SARS-CoV-2 antibody self-test, and subsequently classify results as invalid, IgG negative and IgG positive. A large image library of 595,339 participant-submitted test photographs was created as part of the REACT-2 community SARS-CoV-2 antibody prevalence study in England, UK. Alongside ALFA, we developed an analysis toolkit which could also detect device blood leakage issues.

**Results:**

Automated analysis showed substantial agreement with human experts (Cohen’s kappa 0.90–0.97) and performed consistently better than study participants, particularly for weak positive IgG results. Specificity (98.7–99.4%) and sensitivity (90.1–97.1%) were high compared with visual interpretation by human experts (ranges due to the varying prevalence of weak positive IgG tests in datasets).

**Conclusions:**

Given the potential for LFIAs to be used at scale in the COVID-19 response (for both antibody and antigen testing), even a small improvement in the accuracy of the algorithms could impact the lives of millions of people by reducing the risk of false-positive and false-negative result read-outs by members of the public. Our findings support the use of machine learning-enabled automated reading of at-home antibody lateral flow tests as a tool for improved accuracy for population-level community surveillance.

## Plain language summary

During the COVID-19 pandemic, antibody test kits, for use at home, were used to estimate how many people had COVID antibodies. These estimations indicated how many people have been exposed to the virus or have antibodies due to vaccination. However, some positive test results can be very faint, and be mistaken as negative. In our work, 500,000 people reported their antibody test results and submitted a photograph of their test. We designed a computerised system—a highly specialised artificial-intelligence (AI) system—that has high agreement with experts and can highlight potential mistakes by the public in reading the results of their home tests. This AI system makes it possible to improve the accuracy of monitoring COVID antibodies at the population level (e.g. whole country), which could inform decisions on public health, such as when booster vaccines should be administered.

## Abstract (web summary)

Wong et al. describe a machine learning approach for visual auditing of lateral flow tests for SARS-CoV-2 antibodies. Their automated analysis shows strong agreement with experts and consistently better performance than non-expert study participants at classifying positive results.

## Introduction

Most people infected with SARS-CoV-2, the virus that causes coronavirus disease 2019 (COVID-19), mount an Immunoglobin G (IgG) antibody response detectable after 14–21 days although levels may start to wane after about 90 days^1,2^. As such, antibody data provide a long-lasting measure of SARS-CoV-2 exposure, enabling analyses of the recent epidemic. Indeed, many seroprevalence surveys for SARS-CoV-2 have been reported^3–8^, including our own community-based antibody prevalence study REACT-2 (REal-time Assessment of Community Transmission-2)^9^. REACT-2 is a series of cross-sectional national surveys using home-based unsupervised self-administered lateral flow immunoassay (LFIA) tests for SARS-CoV-2 IgG among random population samples of 100,000–200,000 adults over 18 years in England, UK^9^. While LFIAs may not currently be accurate enough for individual-level diagnosis of SARS-CoV-2 antibody status^10^, on a population level, by adjusting for the sensitivity and specificity characteristics of the LFIA used, it is possible to estimate the levels of past SARS-CoV-2 infection in the community^11^. Therefore, self-conducted LFIAs done at home offer a means to obtain community-wide antibody prevalence estimates rapidly and at reasonable cost.

In general, LFIAs for SARS-CoV-2 antibody collect a small drop of fingerprick blood in a sample well. A reagent buffer is then added to enable the sample to flow up a paper strip. One or two coloured lines (indicating positive, negative, or invalid results) then appear and are read after 10–20 min. Depending on the LFIA, either total antibodies or IgG and Immunoglobin M (IgM) antibodies are detected. Although the LFIA used in REACT-2, Fortress Diagnostics (Northern Ireland), had separate indicator lines for IgG and IgM, only IgG lines were used to estimate the cumulative community prevalence of IgG antibodies for SARS-CoV-2 as part of the study. The initial laboratory validation of the Fortress LFIA was conducted among healthcare workers^12^. It was evaluated as having sensitivity 84.4% (95% CI: 70.5%, 93.5%) and specificity 98.6% (97.1%, 99.4%) compared to “gold standard” ELISA detection of SARS-CoV-2 antibodies^12^. Further usability and acceptability studies were conducted among public-facing non-healthcare key workers^13^ and a random sample of adults in the population^14^. These studies demonstrated high acceptability and usability of self-test LFIAs, including submitting a photograph of the test result to an online portal^13,14^. A review of uploaded photographs of completed tests demonstrated substantial concordance between participants and clinician-interpreted results^13,14^. For home-based self-testing by members of the public there was 93.9% agreement for positive IgG test results, 97.0% for negative, and 98.4% for invalid tests^14^.

However, it is not practical in large population-wide studies, such as REACT-2, for a human expert to review LFIA results at scale. There is a need for a more efficient approach to monitoring for accuracy of responses, including error and subjectivity in participants’ interpretation of test results and systematic defects in batches of devices used. Machine learning models have been shown to demonstrate at least equivalent human performance in a range of diagnostic settings, including interpretation of clinical imaging^15–17^ although there still remain challenges to applying these models to these tasks^18,19^. Recent studies have applied deep learning to the interpretation of human immunodeficiency virus (HIV) field-based rapid diagnostic tests^20^ and SARS-CoV-2 rapid antigen and antibody tests in the form of lateral flow devices^21,22^. However, although these previous studies have demonstrated the feasibility of using artificial intelligence (AI) to improve rapid diagnostic test result interpretation, they were applied to much smaller image libraries (range 3344^22^ to 115,316^21^) vs over 500,000 in our study. In addition, these previous studies involved image capture and test result interpretation by trained healthcare workers or a limited number of “trained operatives”, whereas the REACT-2 study is based on non-expert community testing by members of the public using a variety of capture devices^23^.

Here, we describe the development of a machine learning pipeline (ALFA - Automated Lateral Flow Analysis) to support visual auditing of home-based LFIA self-test results for population-level community SARS-CoV-2 antibody prevalence studies. In applying ALFA to over half a million images, we found excellent agreement between self-readings by members of the public and ALFA’s outputs. When compared to human-expert assessments, ALFA is, however, able to interpret lateral flow read-outs to a level that is, on average, more accurate than untrained members of the public. In the context of mass testing, ALFA, and similar pipelines, have the potential to improve on the assessment of population immunity status via public serological testing at home. Perhaps more importantly, disagreements between ALFA readings and self-read results can be used to flag images for human expert review when used at scale. When millions of tests are to be performed, this is the only practical approach to identify sources of error, which can then inform improvements in user instructions, guidelines, or LFIA device design and manufacture.

## Methods

### REACT-2 antibody prevalence study design and recruitment

**Fig. 1:**
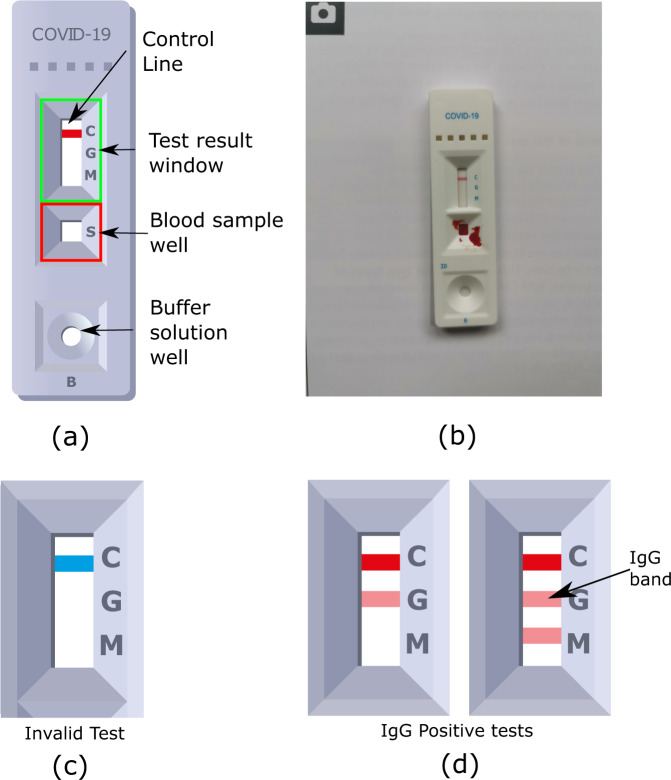
Key visual features of the Fortress SARS-CoV-2 Lateral Flow Immunoassay (LFIA) device. **a** Diagram illustrating the test result window and blood sample well. This diagram shows a negative Immunoglobin G (IgG) test. **b** Example of a participant-submitted photographic image of a negative IgG test. **c** The result window has an initially blue control line, which will remain if the test is unsuccessful (Invalid). **d** In a successful test, the control line turns red, and if IgG antibodies are present in the blood sample, a secondary line will appear below the control. The tertiary line indicates the presence of Immunoglobin M (IgM) antibodies, which were not used as part of the analysis in the REACT-2 study.

The REACT-2 study protocol has been published^9,23^. In summary, REACT-2 is a series of non-overlapping cross-sectional population surveys of the prevalence of SARS-CoV-2 antibodies in the community in England, UK. REACT-2 rounds 1 to 5 were conducted at 1- to 2-month intervals between June 20th 2020 and February 8th 2021 (Supplementary Notes 1, Table S1). At each round, we contacted a random sample of the population by sending a letter to named individuals aged 18 or over from the National Health Service (NHS) patient list (almost the whole population). We then sent respondents a test kit and instructions by post, as well as a link to an online instructional video. The test kits dispatched included 1 LFIA device (Fig. 1), 1 bottle of buffer solution, 1 alcohol wipe, 2 pressure-activated 23 G lancets, and a 1 mL plastic pipette. We asked participants to perform the test at home and complete a questionnaire, including reporting their test results. They were also asked to take a photo of the LFIA on completion of the test and submit a photograph of their completed test via an online portal using instructions and a provided template. The instructions on how to capture the image were designed by the study team and iterated following extensive involvement of the REACT Public Advisory Group in order to improve the quality and standardisation of image capturing by participants. Survey instruments, including the instruction booklet and images of the test kit components, are available on the study website (https://www.imperial.ac.uk/medicine/research-and-impact/groups/react-study/).

### REACT-2 LFIA image library

Across Rounds 1–5, there were 740,356 participants of whom 81.7% (605,013) submitted images available for this study. After stripping participants’ personal identifiers, including geocoordinates, images were stored on a secure server managed by Imperial College London. We were able to use, and thus analyse, 98.4% (595,339) of these images (Supplementary Notes 1, Table S1). Images could not be used if the LFIA device or test result window were not present in the image, or due to image corruption/error (<2%). Subsets of this image library were used to train and improve the machine learning algorithms for high concordance with visual interpretation by human experts (clinical scientists with experience of reading LFIAs) and for optimising sensitivity and specificity. The subsets are detailed in Table 1. Some samples (from Round 5) of the subsets were selected as they fulfilled a certain criterion (having been submitted by participants who were vaccinated 21+ days prior, and self-reported as a negative result).

**Table 1:** A breakdown of the subsets used to develop ALFA.

Dataset	1		2	3		4	
Experiment	Segmentation Network		Validity (Control-line status)	IgG Status Classification 1		IgG Status Classification 2	
Purpose	Develop	Test	Test	Develop	Test	Develop	Test
No. of samples	415	83	187	864	294	1699	237
No. of IgG positives	–	–	–	351	143	641	79
No. of invalids	–	–	12	–	–	–	–
The subsets from REACT-2 Study-5 Rounds which were combined to form the datasets (“Included” indicates inclusion)							
Round-1, Sample A	Included		–	Included		Included	
Round-1, Sample B	Included		Included	Included		Included	
Round-1, Sample C	–		–	Included		Included	
Round-2 sample	–		–	Included		Included	
Round-5 sample	–		–	–		Included	

“Experiment” refers to which component of ALFA was being developed/tested. Most datasets were split into development and test subsets and indicated in the “purpose” row. The origins of the images are also shown in the bottom half of the table. “Round” refers to the round of the REACT-2 Study-5 from which the data were collected. “Included” indicate whether the sample was included in the dataset.

### ALFA pipeline design and development

The methods and pipeline are described in full in the Supplementary Methods of Supplementary Notes 1 and 2. The ground truth used for the developing and testing of our computational pipeline, ALFA, was obtained from expert manual review and labelling of the LFIA images. Two experts labelled the images to identify key regions of the flow device, and to assess the validity and IgG status of the LFIA result, with a third independent reviewer brought in for cases of disagreement in the latter. Experts did not have access to the original LFIA tests in the images and the LFIA did not have an accompanying gold-standard test, for example, ELISA detection of SARS-CoV-2 antibodies. Human experts were also blinded to the participants’ submitted interpretation of the LFIA result.

Supplementary Notes 1, Fig. S1 illustrates the workflow of the ALFA pipeline which takes participants’ images of LFIAs captured at the time of reading the test result, and the corresponding participants’ result interpretations as input. The first pre-processing step (A) uses a deep convolutional neural network (CNN) to identify candidate regions of interest (ROIs) in the participant-submitted images. These regions are (i) the LFIA itself, (ii) the test result (read-out) window, and (iii) the blood sample well (Supplementary Notes 2, Supplementary Methods). Using geometric priors, as detailed in Supplementary Notes 1, Table S2, we report segmentation failures, including erroneous and poor-quality images, and remove them before further analysis as a form of quality control. The images are then rotated so that the read-out windows are orientated in a consistent manner, this step is shown in Supplementary Figure S2. The second step (B) involves uses a combination of signal processing, computer vision and machine learning to obtain an interpretation of the LFIA result. Details of the algorithm chain are in Supplementary Notes, Supplementary Methods (Figs. S3, S4, and S5). To obtain informative samples^24^ for machine learning, three phases of development were used to read the IgG status for valid tests. Discrepancies between participant and early versions of the algorithm (Phase 1) in early rounds of REACT 2 were used to identify difficult cases for training small networks in Phase 2, and these, in turn, used to identify difficult cases for training larger networks in Phase 3. Finally, in the third step (C) a comparison between the users and ALFA’s interpretation is completed. Disagreements are reported and are reviewed for later inspection. Details on the networks and training can be found in Supplementary Notes 2.

Early development and testing of our Phase 2 1D CNN (Architecture represented in Supplementary Table S5) and Phase 3 2D CNN (Based on MobileNetV2^25^) were completed in Classification Experiment 1 (CE1). The training and validation (development) datasets consist of samples from Study-5’s R1 and the test set came from Study-5’s R2, removing risk of data leakage. For CE1, we did five-fold cross-validation on just the development set. After preliminary cross-validation showed promising results with high average sensitivity and specificity, we then retrained the models on the whole development set and reported the performance of the final model evaluated on the test set. The 2D CNN was then deployed into the ALFA pipeline for initial analysis of the full REACT-2 Study-5 dataset of 500,000 images.

A pattern emerged in the Round 5 samples, which consisted of participants who reported seronegative results even after having a vaccination 21 days prior to completing the LFIA. Experts reviewed these cases and found “weak IgG positive” samples which were being miss-classified by the CE1 networks. These weakly positive samples were used to create a new dataset with a larger proportion of weak positive samples, forming the basis of Classification Experiment 2 (CE2). The aim of this was to improve the sensitivity of the CNNs to weak positives.

For completeness, we included in CE1 and CE2 experiments the results of an initial algorithm (Heuristic-C) based on peak-detection techniques (See Supplementary Notes2’s Supplementary Methods and Supplementary Notes 3) to classify the IgG status of samples; these are used before sufficient data were manually labelled to support the machine learning. A full comparison detailing other 1D CNN architectures are also available in Supplementary Tables S6 and S7.

After optimizing the algorithms to maximise the likelihood of correctly classifying an LFIA test result, and having evidence of high agreement between traditional visual inspection by human experts and ALFA, we were able to deploy the pipeline to analyse over 500,000 REACT-2 images to assess the performance of the REACT-2 study participants as well as generating potential estimates for the population’s SARS-CoV-2 antibody prevalence. The use of two-dimensional Convolutional Neural Networks (2D CNNs) for LFIAs and a new image capture protocol builds on research by the McKendry group and the i-sense interdisciplinary research collaboration (IRC) (www.i-sense.org.uk) at University College London^20^.

### Performance indicators

The four indicators of performance were sensitivity, specificity, total accuracy (ALFA correctly classified LFIA test results against human expert review) and Cohen’s kappa. The ground truth for comparison was the visual interpretation of LFIA test result images by two human experts.

### Sensitivity, specificity, and accuracy

1 $${{{{{{\rm{Specificity}}}}}}}=\frac{{{{{{{\rm{True}}}}}}}\,{{{{{{\rm{negative}}}}}}}}{{{{{{{\rm{True}}}}}}}\,{{{{{{\rm{negative}}}}}}}+{{{{{{\rm{False}}}}}}}\,{{{{{{\rm{positive}}}}}}}}$$

2 $${{{{{{\rm{Sensitivity}}}}}}}=\frac{{{{{{{\rm{True}}}}}}}\,{{{{{{\rm{postive}}}}}}}}{{{{{{{\rm{True}}}}}}}\,{{{{{{\rm{positive}}}}}}}+{{{{{{\rm{False}}}}}}}\,{{{{{{\rm{negative}}}}}}}}$$

3 $${{{{{{\rm{Accuracy}}}}}}}=\frac{{{{{{{\rm{True}}}}}}}\,{{{{{{\rm{postive}}}}}}}+{{{{{{\rm{True}}}}}}}\,{{{{{{\rm{negative}}}}}}}}{{{{{{{\rm{Total}}}}}}}\,{{{{{{\rm{no}}}}}}}.\,{{{{{{\rm{of}}}}}}}\,{{{{{{\rm{samples}}}}}}}}$$

### Cohen’s kappa

4 $$\kappa =\frac{{\rho }_{o}-\,{\rho }_{e}}{1-\,{\rho }_{e}}$$

where ρ_o_ is the relative observed agreement between method (or participants) and ground truth, and *ρ*_e_ is the hypothetical probability of chance agreement,

5 $${\rho }_{e}{{{{{\boldsymbol{=}}}}}}\frac{1}{{N}^{2}}\mathop{\sum}\limits_{c}{n}_{c1}{n}_{c2}$$

where *n*_*ci*_ is the number of times rater *i* (i=1,2) predicted category *c* among *N* observations.

Interpretation of Cohen’s kappa values is as follows: <0 poor agreement, 0.00–0.20 slight agreement, 0.21–0.40 fair agreement, 0.41–0.60 moderate agreement, 0.61–0.80 substantial agreement, and >0.8 almost perfect agreement^26^.

### Ethics

Ethical approval was granted by the South Central-Berkshire B Research Ethics Committee (IRAS ID: 283787). Participants provided informed consent when they registered for the study (via electronic consent). Electronic consent was recorded by a tick box. This method of consent was approved by the ethics committee. All data were handled securely in accordance with a detailed privacy statement.

## Results

Automated analysis showed “substantial agreement” with human experts and the 2D CNNs performed consistently better than study participants in terms of accuracy and Cohen’s kappa, particularly for weak positive IgG results (Tables 2 and 3, CE1 and CE2 results, respectively). In experiments using two datasets with substantially different proportions of weak positives (CE1, CE2), Cohen’s kappa for machine read-outs of IgG status against human experts ranged from 0.905 (95% CI: 0.847–0.962) for the set with more weak positives (Table 3), to 0.966 (95% CI: 0.936–0.996) with fewer weak positives (Table 2). Specificity ranged from 0.987 (95% CI: 0.968–0.999) to 0.994 (95% CI: 0.979–0.999), whilst sensitivity ranged from 0.901 (95% CI: 0.837–0.950) to 0.971 (95% CI: 0.940–0.991) for detecting IgG status (Tables 2, 3). When the proportion of weak positives is lower (Table 2), the participants have better sensitivity than the 2D CNN, but lower specificity. Once the proportion of weak positives increases (Table 3), we can see that the 2D CNNs sensitivity is now greater than the participants while maintaining higher specificity.

**Table 2:** Results of classification experiment 1 (CE1) (*N* = 294, dataset 3’s test dataset with fewer weak positive results).

Model/heuristic/participants	Specificity	Sensitivity	Accuracy	Cohen’s kappa
2D CNN	0.994	0.971	0.983	0.966
1D CNN	0.999	0.879	0.941	0.882
Heuristic-C	0.994	0.757	0.881	0.759
Study participants	0.961	1.000	0.980	0.959

*CNN* Convolutional Neural Network.

**Table 3:** Results of classification experiment 2 (CE2), including the performance of the 2D CNN trained in experiment 1 (CE1), on dataset 4’s test set with more weak positive results (*N* = 237).

Model/heuristic/participants	Specificity	Sensitivity	Accuracy	Cohen’s kappa
2D CNN (CE1)	1.000	0.852	0.949	0.883
2D CNN (CE2, retrained)	0.987	0.901	0.958	0.905
1D CNN	0.968	0.844	0.926	0.733
Heuristic-C	0.976	0.487	0.815	0.525
ViT Net	0.968	0.952	0.962	0.917
Study Participants	0.974	0.679	0.873	0.699

*CNN* Convolutional Neural Network.

These results highlight the necessity of providing difficult cases in the training data. As the 2D CNN model trained in CE1 attained (jointly) the best sensitivity and specificity, we applied it to the test set of CE2. The results show that CE1 version of the 2D CNN performed poorly in detecting weak positives; however, after retraining the model (CE2, retrained), we showed improvement. The 2D CNN (CE2, retrained) was the overall best performing model in a dataset that contains a larger number of weak positive results. A Visual Transformer Network^27^ was later trained for this task and achieved better sensitivity, accuracy and Cohen’s kappa. However, it was decided further work was required before implementing it into ALFA due to the lower specificity than the 2D CNNs; we have made this network available on https://github.com/TianhongDai/react2-code, given its performance in our dataset.

**Fig. 2:**
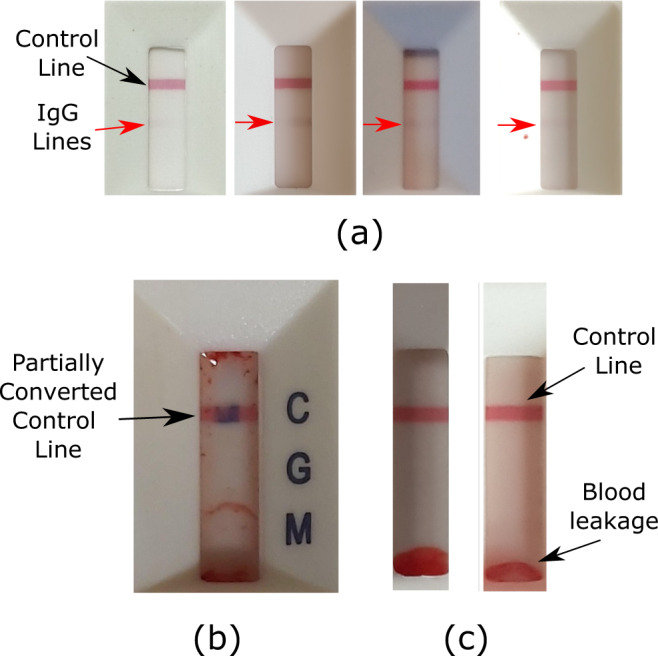
Examples of issues with the Lateral Flow Immunassay (LFIA) device. **a** Examples of weak Immunoglobin G (IgG) positive LFIA results. The control line is blue if the test has not been completed, turning red if the sample and buffer have been correctly added. ‘Weak’ IgG positives were highlighted as being a challenging scenario for the algorithms to classify correctly. As seen in the examples, the line is faint, and with additional issues of variable lighting. The solution was to introduce more of these cases into the training data. **b** An invalid LFIA test with a partially converted control line. The method for determining validity looked at the normalised red and blue pixel intensity at the detected control line. A potential source of misclassification is partial conversion of the control line from blue to red. **c** Examples of blood leakage on the LFIA. The presence of blood leakage at the sample and buffer end of the read-out window was found to be a common source of misreading by participants. This led many participants to report the test result as being Immunoglobin M (IgM) positive even though the test was both IgG and IgM negative.

Regarding detecting invalid tests, the current best performing algorithm had a sensitivity of 0.917 (95% CI: 0.760–0.990), and a specificity of 1.000. Potential sources of error are primarily due to partially converted control lines (Fig. 2b).

**Fig. 3:**
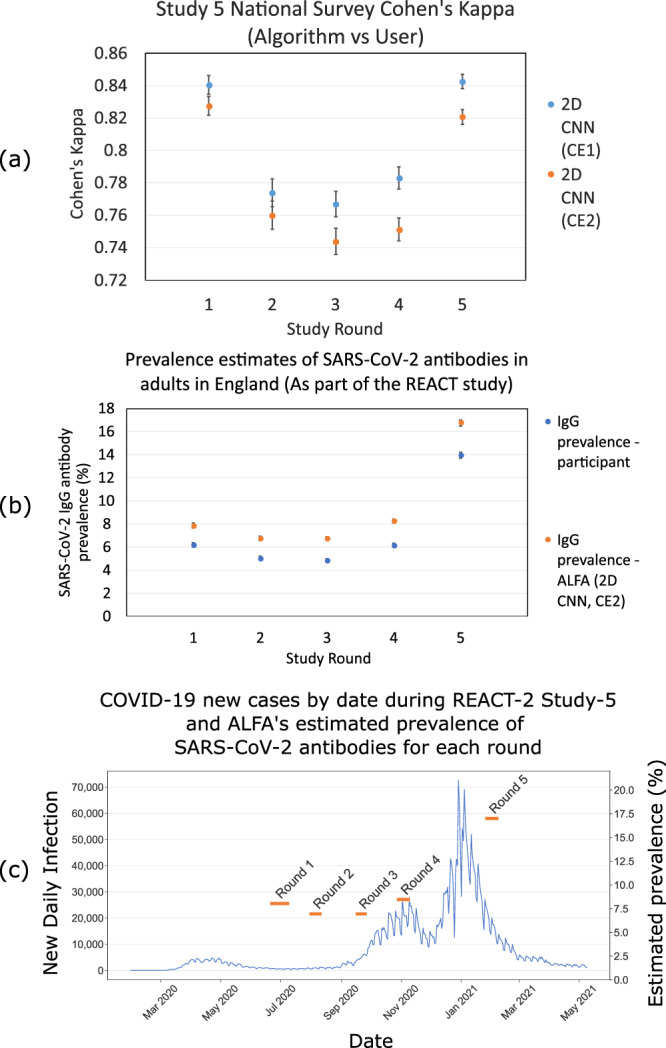
Comparison between automated lateral flow analysis (ALFA) pipeline and REACT-2 Study-5 participants. **a** Cohen’s kappa between the two 2D Convolutional Neural Network (CNN) algorithms developed as part of the ALFA pipeline and study participants for REACT-2 rounds 1 to 5 (Respectively for each round, *N* = 93252, *N* = 95508, *N* = 123614, *N* = 133225, *N* = 140240). 2D CNN (Classification Experiment 2 (CE2), Blue) and 2D CNN (Classification Experiment 1 (CE1), Orange) indicate strong Cohen’s kappa, suggesting that participants are very good at reading their own results throughout the series of surveys. 2D CNN (CE1) is likely to have higher agreement than 2D CNN (CE2) because CE2 is picking up a greater proportion of weak positives, some of which are being missed by participants. The ALFA pipeline suggests substantial agreement between (0.70) participants’ readings and the ALFA pipeline, providing confidence in the self-reading of fingerprick blood home self-testing LFIA kits. Error bars represent a 95% confidence interval. **b** Estimated prevalence of SARS-CoV-2 Immunoglobin G (IgG) antibodies in adults in England in the REACT-2 study by i) participant-reported results (Blue) and ii) ALFA (2D CNN CE2, Orange) automated read-out. Error bars represent 95% confidence interval, respectively for each round, *N* = 88557, *N* = 94291, *N* = 122211, *N* = 131327, *N* = 138200. **c** ALFA (2D CNN CE2, Orange bars) estimated antibody prevalence (Respectively for each round, *N* = 88557, *N* = 94291, *N* = 122211, *N* = 131327, *N* = 138200) overlaying daily new test positive COVID-19 cases (Blue line graph, Data acquired from GOV.UK here: https://coronavirus.data.gov.uk/).

We compared self-reported participant readings with ALFA read-outs over all interpretable images of the REACT-2 study rounds 1 to 5 (*N* = 595,339) using Cohen’s kappa to quantify the participants’ ability to interpret LFIA results. We found a Cohen’s kappa of 0.797 (95% CI: 0.794–0.799) between participants’ and ALFA read-out of test validity and IgG status (Fig. 3a shows the kappa values for individual rounds. Disagreements occurred primarily when the algorithm reported an IgG positive test and study participants submitted a negative result.

A hypothesis for the slightly fluctuating level of Cohen’s kappa between rounds is the following: many discrepancies between ALFA and the interpretation of a particular participant are due to weak positive IgG lines being detected by ALFA, but missed by the participant. The number of these weak positives will vary depending on the background community antibody prevalence, which we know varied over the course of the study. For example, Round 3, which returned the lowest Cohen’s kappa, and therefore contained the largest proportion of discrepancies between the ALFA pipeline and study participants in determining positive results, coincided with increasing daily SARS-CoV-2 cases. During this period, there is likely to have been a larger proportion of participants who were recently infected (it takes 2–3 weeks to develop IgG antibodies post-infection^3^) and will not have fully developed the IgG antibody responses. Hence, a substantial number of weak positives could be expected during the Round 3 study interval. Additionally, participants who possibly were infected earlier in the pandemic could have waning antibodies, adding another source of weak positives. For Rounds 4 and 5, which coincided with the peak of the 2nd wave of the pandemic, we saw that the value of Cohen’s kappa increased. Following our hypothesis, this would be due to weak positives making up a smaller proportion of the number of positive samples.

Alongside ALFA, we developed a toolkit which also detected device blood leakage issues using projection signatures (signatures are explained in Supplementary Notes 3, Supplementary Methods). The toolkit analyses ensembles of participant LFIA responses generated from their LFIA images, allowing identification of trends in responses accumulated over thousands of participants in addition to the individual readings provided by participants. By using this approach, it was possible to identify a consistent source of error in participants’ interpretation of a specific assay component of the LFIA device. Specifically, we identified a tendency to misread the results when blood exhibited a particular form of leakage pattern on the LFIA (Fig. 2c). The methods are described in full in Supplementary Notes 4.

## Discussion

We have developed a computational pipeline (ALFA) that can accurately classify the results of the Fortress LFIA for SARS-CoV-2 antibody detection based on test result images taken and submitted by over 500,000 participants as part of the REACT-2 community SARS-CoV-2 antibody prevalence study, the largest study of its kind worldwide^9^. We demonstrated the potential of the pipeline to accurately classify LFIA test result images, with an overall performance comparable to that of a human expert and slightly higher than that of a study participant, particularly for weak positive IgG results. This suggests that the ALFA pipeline can support the identification of mistakes in reading made by study participants, and as such reduce the number of false positives and false negatives. At low population SARS-CoV-2 IgG prevalence, as was seen in the UK prior to high vaccine coverage but which is also the most likely scenario in countries at earlier stages in their vaccination programmes, the observed higher specificity of the pipeline compared to participants will produce less false positives. This is important for surveillance in low-prevalence settings so as not to produce overestimates, and as such, false reassurance with regards to population antibody levels. Reducing false positives is also beneficial in terms of how individuals may respond to knowing their antibody status, as an individual who has an IgG positive test may feel less at risk of severe disease, and change behaviour accordingly. Higher sensitivity of the pipeline, particularly for weak positive IgG results, is an advantage in high prevalence settings so as not to underestimate the impact of COVID-19 vaccination programmes on population antibody levels, and therefore to better reflect their success.

The use of the pipeline in REACT-2 has already played a critical role in supporting the assessment of the accuracy of visual interpretation of the self-test by study participants. Based on ALFA’s ability, compared to REACT-2 participants, to better detect weak positives, population seroprevalence estimates could become more accurate, particularly during periods of low exposure and waning immunity when the prevalence of weak positives is likely to be greater. Additionally, analysis of projection signatures generated from LFIA read-out windows (Supplementary Notes 4, Figs. S6, S7, S8) yielded a simple mechanism to detect blood leakage (Supplementary Fig. S7) into the read-out window, another potential source of misinterpretation by participants. On the face of it, LFIAs seem easy to read. However, we have demonstrated that it is possible to obtain seroprevalence read-outs that are more accurate than an untrained lay member of the public in test result interpretation, thus lower reading uncertainties, and in uncontrolled capture conditions. This creates opportunities for patient self-testing for community serological surveillance and determining antibody status for SARS-CoV-2 without having to have human experts manually interpret or check test results. So, in terms of time and cost, the savings lie in the expert human resources required to check results manually.

Our results support earlier acceptability and usability studies undertaken as part of the REACT-2 programme which concluded that participants’ ability to correctly read IgG status from the LFIA device was very high but disagreements were most likely due to weak positives being misread by participants as negative^13,14^. In addition, other studies exploring the use of deep learning algorithms for automated reading of LFIAs have demonstrated good performance, including a reduced number of false positives and false negatives^20–22^. Turbé et al.^20^ used deep learning algorithms to classify images of LFIAs for the identification of HIV and demonstrated high levels of accuracy (98.9%) compared with visual interpretation by community health workers (92.1%) using a human expert LFIA interpreter as the gold standard, in rural South Africa^20^. Specifically, for COVID-19, a study exploring the performance of a smartphone application that used machine learning to classify SARS-CoV-2 serological results across 11 LFIA devices showed high sensitivity (98.9%) and specificity (99.7%) compared to reading by a human expert^22^. Although the Fortress LFIA was not part of that study, it also found the same blood leakage issues with other LFIA devices that we detected with the Fortress device. Finally, a UK study on lateral flow devices for SARS-CoV-2 antigen detection using visual interpretation by healthcare workers staffing asymptomatic test sites in the community, and healthcare workers undertaking at-home self-testing found that the use of an AI algorithm based on machine learning outperformed the participants^21^.

Like the studies reported by Beggs et al.^21^ and Mendels et al.^22^, we used multiple networks that split the task of image analysis. Indeed, our recommendation is that a single network is not the right approach. One needs a combination of computer vision and machine learning to achieve state-of-the-art results without exorbitant degrees of manual labelling. Though the number of images analysed in our study is unprecedented, we note that a previous study of LFIAs for SARS-CoV-2 antibody detection also used another network to classify the device type^22^. This is a good idea, and we would suggest that a good global policy would be to establish a database of device images, spanning multiple device types and manufacturers to enable classification to be done. Pre-trained models for interpretation of specific lateral flow tests can then be retrieved and applied as appropriate.

Although previous studies demonstrate the feasibility of using AI to improve rapid diagnostic test result interpretation, the photographs of the devices were taken by healthcare workers or technicians who had training^20,22^. For example, in the blood serum study reported by Mendels et al.^22^, the lateral flow device was mounted using a stand at a fixed distance from the capture device with controlled lighting; in contrast, the REACT-2 study’s database consists of images taken by over 500,000 participants (selected at random from NHS GP lists) with only guidance from an instruction booklet. Previous studies that have utilised participant images^20^ had low population antibody prevalence in their dataset, partly due to the timing of their data collection. In contrast, REACT-2 was conducted over several months, yielding 500,000 images with varying population prevalence. At this scale, we were able to observe a larger number of positive samples, yielding an estimate of AI sensitivity that is more likely to be realistic than prior studies. Additionally, previous work identified “low-contrast” lines (weak positives) that were missed by human readers. This issue was directly addressed in our study through a training process for the read-out component of the ALFA pipeline that incorporated training examples of weakly positive LFIA results; these samples were identified by expert review of discrepancies in LFIA reading between vaccinated participants of REACT-2 and the results produced by an early iteration of pipeline development. Once trained on these samples, the ALFA pipeline was better than participants at reading weak positive results (detecting faint test lines), thus reducing false negatives, and mirroring earlier findings^21^. Table 4 summarises the differences between our study (Wong et al.) and previous works. Alongside the differences in scale and image acquisition, there were also marked differences in the devices and biological samples used. For example, the AI LFD Consortium^21^ developed the AI and analysed performance for COVID-19 antigen tests, which use a clear biological sample (nasal/throat fluid) and therefore the read-out does not have the visual complexity of a LFIA associated with blood serum samples due to potential blood splatter and leakage (see Supplementary Notes 4, Fig. S7).

**Table 4:** A comparison to show the advances of this study (Wong et al.) over other recent research.

	Sample/LFIA Target	Tablet device (single device type)	Study Participants	Technology	Train-Test Set	Deployment
Turbe et al.^20^	Blood Serum/HIV antibody	Tablet device (single device type)	60 Field Workers	*MobileNet v2*	11,374	40 tests in the field
LFD AI Consortium (Beggs, corr author)^21^, Lancet, 2021.	Nasopharyngeal Swab/ SARS-CoV-2 antigen	Expert Operator + participant at-home testing	Asymptomatic, provider-led community testing and NHS workers at home.	Multiple networks	115,316	Unknown
Mendels et al.^22^	Blood Serum/SARS-CoV-2 antibody	Custom Stand with fixed distance/pose to camera; 3 image capture types, multiple LFIAs.	Not specified	Multiple networks	4344	N/A
Wong (this paper)	Blood Serum/ SARS-CoV-2 antibody	Hand-held camera, multiple capture types, untrained users, multiple LFIA types.	Lay population self-testing	Multiple networks	1936	500,000+ in the field

Practically speaking, we believe that the framework of the ALFA pipeline is translatable to other home testing scenarios. It will be necessary to retrain segmentation networks for different lateral flow test devices, but our experience has been that this is easily done in a single round of home data collection, requiring only markup of salient regions. Projecting from our results, the training of the read-out algorithm to high sensitivity requires at least 3000 samples, with a strong representation of expertly labeled weak-positive examples. Flagging such samples at the start is therefore important. Such cases can be identified through examining discrepancies in early versions of deployment, and adding them to the training set as “difficult” cases to create the next version of classifier: one should expect and plan for such iteration to be part of the process. Periodic retraining of the read-out network is also advisable; we suggest that such periodic review and training may be triggered by detecting changes in Cohen’s kappa between users and AI system results. Other skillsets for teams seeking to implement similar systems include AI systems architecture, but this should be achievable by IT teams familiar with electronic public polling.

There are limitations to our study. We have developed the algorithms to have high sensitivity for IgG positive samples and achieved performance on par with human expert visual audits. However, the current deployed version of ALFA does not specifically handle potential sources of error such as slight blurring of an image, LFIA having blood leakage, LFIA defects or lighting issues. Given any network may produce unexpected outputs if the data presented to it is out-of-distribution of its training data, this is still a risk. Future work will look at utilising the signature analysis toolkit to collate training examples of these “anomalies” from the full REACT dataset and train a network which is more comprehensive at interpreting specific anomalies in the submitted images.

Since the key aim of this work was to acquire image data to develop, fully characterise AI accuracy, and to analyse user-errors in conducting tests, the ALFA functionality has not been engineered into a mobile “App”. Being “App”-free reduces user-facing support costs in deployment, since the only device functionality that is required is image upload; this broadened the range of participants, some of whom might be wary of downloading an App, or may not have smartphone access (the sociodemographic characteristics of REACT-2 Study 5 are noted in Supplementary Notes 1, Supplementary Discussion, Table S3 and S4). Indeed, we noted that Webcams or tablets were used to acquire and upload some results. Focusing on the back-end pipeline also provided the opportunity to gather information on the types of errors that may be encountered in acquiring images, which is useful at this stage of development; outcomes of analysis of user vs algorithm interpretation will provide insight into how to select upload resolution, and provide on-device guidance for future App-based image acquisition systems, and eventually robust App-based readers.

To our knowledge, with over 500,000 test images in our library, we have conducted the world’s largest study exploring the potential of machine learning algorithms to read the results of LFIAs performed at home by members of the public. The primary use case for the pipeline is in reviewing the accuracy, at the population level rather than the individual level, of participant-reported LFIA test results for estimating SARS-CoV-2 antibody prevalence. Its application could be to identify and examine trends that may highlight sources of systematic error or device failure. These can then inform improvements to study processes or user instructions. Given the potential for LFIA devices to be used at scale as part of the COVID-19 response, even a small improvement in accuracy could reduce the risk of false positive and false negative result read-outs by members of the public. The benefits include more accurate estimates of community prevalence of SARS-CoV-2 IgG antibody, which will be critical for supporting policy decisions regarding future COVID-19 vaccination campaigns. Indeed, we have already used the pipeline in Round 6 of the REACT-2 study to produce more accurate estimates of seroprevalence. Based on participant read-outs, the estimated community IgG prevalence in England, adjusted for the performance characteristics of the test and weighted to the population, was 61.1%^28^. The equivalent estimate using the ALFA read-out was 66.4%. In addition, if future vaccination policy is based on an individual’s antibody status, lower reading uncertainties by providing individuals and clinicians with more accurate automated readings of at-home self-testing LFIAs will support better decision-making regarding whom and when additional vaccine doses should be offered. In this regard, we expect such a system for automated reading to enable support of members of the public who are not confident in interpreting test results from LFIA devices. The computational pipeline could be adapted to support a similar function for LFIAs used for SARS-CoV-2 antigen detection. The considerations in this particular work are specific to the Fortress device for SARS-CoV-2 antibody testing. However, the ALFA pipeline is modular, and elements can be adapted and adjusted in sequence for new devices, including LFIAs used in the diagnosis and surveillance of other diseases.

### Reporting summary

Further information on research design is available in the Nature Research Reporting Summary linked to this article.

## Supplementary information

Supplementary Data 1

Supplementary Data 2

Supplementary Data 3

Description of Additional Supplementary Files

Supplementary Information

Reporting Summary

## Data Availability

For the underlying data, access to this data is restricted due to ethical and security considerations. To obtain ethics approval from the South Central Berkshire B Research Ethics Committee (REC) and Health Regulator Authority (HRA), we agreed that we will preserve the confidentiality of participants taking part in the study and fulfil transparency requirements under the General Data Protection Regulation for health and care research. We also agreed that all REACT study data is to be held securely and processed in a Secure Enclave. This is an isolated environment within Imperial College for the processing of health-related personal data. It provides a framework that satisfies Information Governance requirements that come from several sources. The Secure Enclaves are compliant with the requirements of major data providers (e.g. ONS, NHS Digital and NHS Trusts), as well as flexible to incorporate additional requirements a group may be subject to. The enclaves are ISO27001 certified. These restrictions apply to all the study data, both qualitative and quantitative. We do not allow any line list data to be taken from the secure enclave because of the risk of cross-referencing and deductive disclosure. A researcher can request access to the data held in the Secure Enclave by emailing react.access@imperial.ac.uk. Access would be granted to researchers for the purposes of further research subject to approval by the data access committee and after signing a data access agreement to ensure no disclosure of potentially identifying details. Source data for Fig. 3a, b, and c is available in Supplementary Data 1, 2, and 3 respectively. Figure 3c utilises data from Supplementary Data 2 and 3.

## Code availability

Code and additional data to support the figures is freely available on this GitHub repo: https://github.com/TianhongDai/react2-code. The DOI is provided by Zenodo^29^.
