# Supplementary material for: Machine learning to support visual auditing of home-based lateral flow immunoassay self-test results for SARS-CoV-2 antibodies
Source: Commun Med (Lond). 2022 Jul 6;2:78. doi: 10.1038/s43856-022-00146-z (PMC9259560; doi:10.1038/s43856-022-00146-z)
Supplement: Supplementary file 4 — Description of Additional Supplementary Files [file 43856_2022_146_MOESM4_ESM.pdf]

Description of Additional Supplementary Files

File Name: Supplementary Data 1

Description: This is the dataset for Figure 3A. Cohen's kappa for each round between ALFA and ReACT-2 Study-5 participants

File Name: Supplementary Data 2

Description: This is the dataset for Figure 3B and 3C. Contains the prevalence estimates for the Convolutional Neural Networks.

File Name: Supplementary Data 3

Description: This is the dataset for Figure 3C. Contains the daily number of infections during the course of REACT-2 Study-5.
