# Supplementary material for: Machine learning to support visual auditing of home-based lateral flow immunoassay self-test results for SARS-CoV-2 antibodies
Source: Commun Med (Lond). 2022 Jul 6;2:78. doi: 10.1038/s43856-022-00146-z (PMC9259560; doi:10.1038/s43856-022-00146-z)
Supplement: Supplementary file 5 — Supplementary Information [file 43856_2022_146_MOESM5_ESM.pdf]

Machine learning to support visual auditing of home-based lateral flow immunoassay self-test results for SARS-CoV-2 antibodies – Supplementary document

Nathan C K Wong[1*], Sepehr Meshkinfamfard[2], Valérian Turbé[2], Matthew Whitaker[3], Maya Moshe[4], Alessia Bardanzellu[1], Tianhong Dai[1], Eduardo Pignatelli[1], Wendy Barclay[5,4,6], Ara Darzi[5,7,6], Paul Elliott[3,5,6,8], Helen Ward[3,5,6], Reiko J Tanaka[1], Graham S Cooke[4,6], Rachel A McKendry[2,9*], Christina J Atchison[3,5*], and Anil A Bharath[1*]

1 Department of Bioengineering, Imperial College London, London, UK

2 London Centre for Nanotechnology, University College London, London, UK

3 School of Public Health, Imperial College London, London, UK

4 Department of Infectious Disease, Imperial College London, London, UK

5 Imperial College Healthcare NHS Trust, London, UK

6 National Institute for Health Research Imperial Biomedical Research Centre, London, UK

7 Institute of Global Health Innovation, Imperial College London, London, UK

8 MRC Centre for Environment and Health, School of Public Health, Imperial College London, London, UK

9 Division of Medicine, University College London, London, UK

Corresponding Authors: nathan.wong16@imperial.ac.uk, christina.atchison11@imperial.ac.uk, r.a.mckendry@ucl.ac.uk, a.bharath@imperial.ac.uk

Supplementary Notes 1

Figure S1: Flow diagram illustrating the (Automated Lateral Flow Analysis) ALFA pipeline. Rather than using a single network for read out, we split the functions of the pipeline into a series of stages. This facilitates i) interpretation and analytics, ii) detecting out-of-distribution images that might lead to unpredictable results; iii) gradual increase in the sophistication of algorithms as more image data is accumulated. Abbreviations are defined as follows: LFIA (Lateral Flow Immunoassay), QA (Quality Assurance), CNN (Convolutional Neural Network).

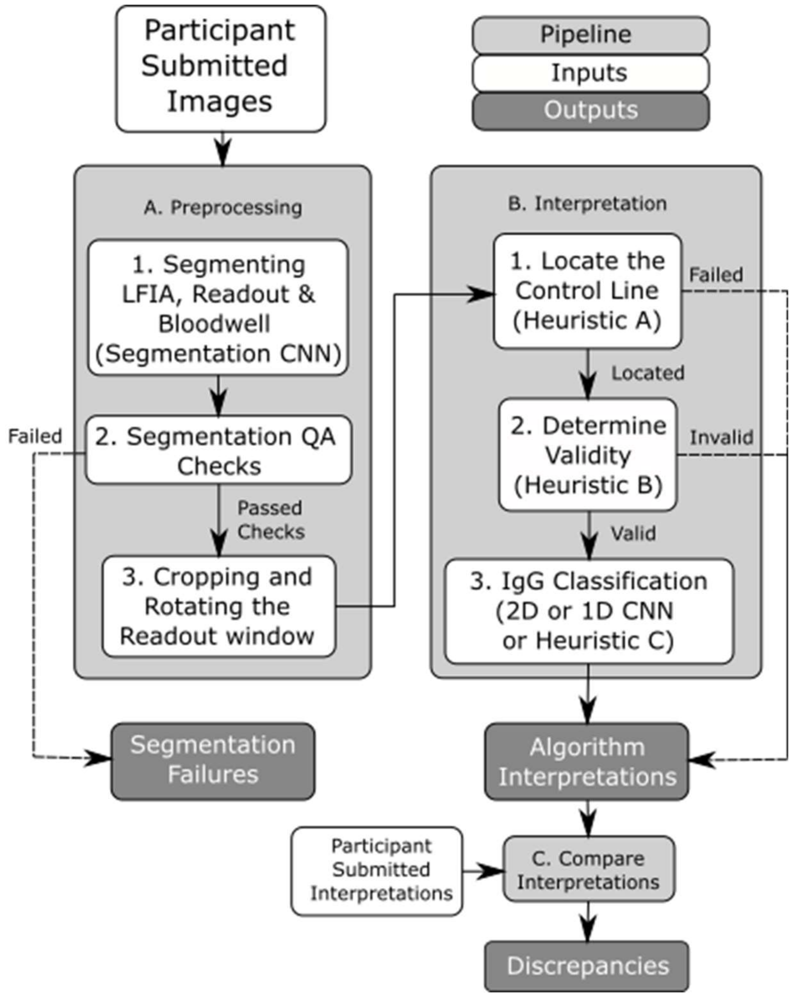

Figure S2: Intermediate steps to the rotating and cropping of the Lateral Flow Immunoassay (LFIA) test result window. a Vector (Blue) from centroid of Region Of Interests. **b** Image post-rotation. **c** Extracted read-out window.

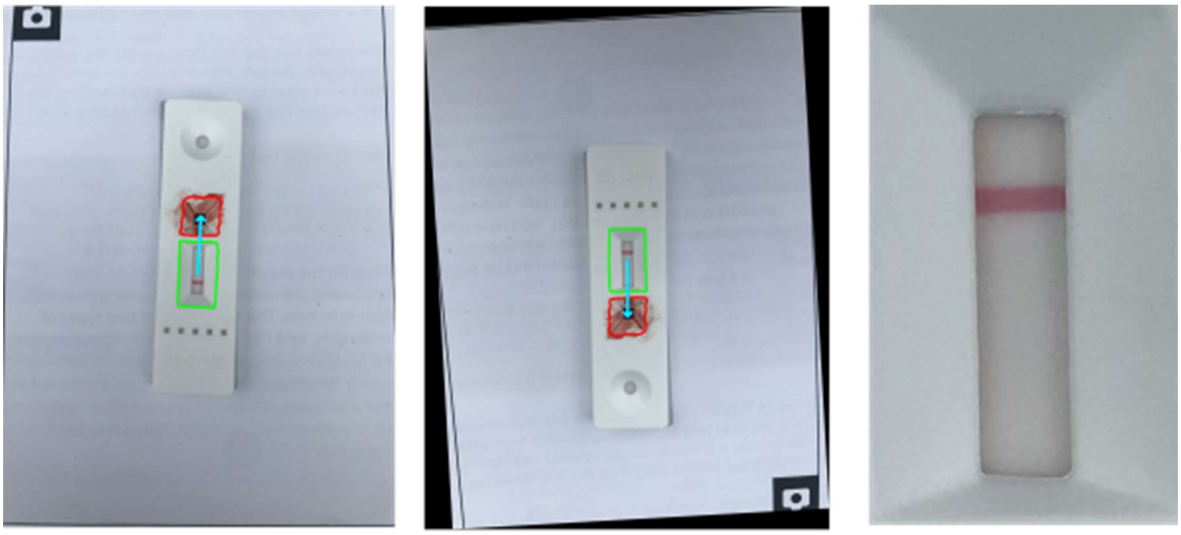

(a) (b) (c)

Figure S3: Examples of projection signatures and Colour spaces. The projection signatures are generated by averaging (mean) the images pixel values across the short axis, which produces a signal in the long axis. **a** Red, green, and blue colour channels, and their projection signatures, $P_{(R|G|B)}(x_\theta)$. **b** Normalised red, green, and blue colour channels, and the signatures $P_{(nR|nG|nB)}(x_\theta)$. **c** Hue, saturation, and value (intensity) channels, and signatures $P_{(H|S|V)}(x_\theta)$. **d** The O opponency channel, and its projection signature, $P_O(x_\theta)$. Abbreviations are defined as follows: normalised red (nRed and nR), normalised green (nGreen and nG), and normalised blue (nBlue and nB).

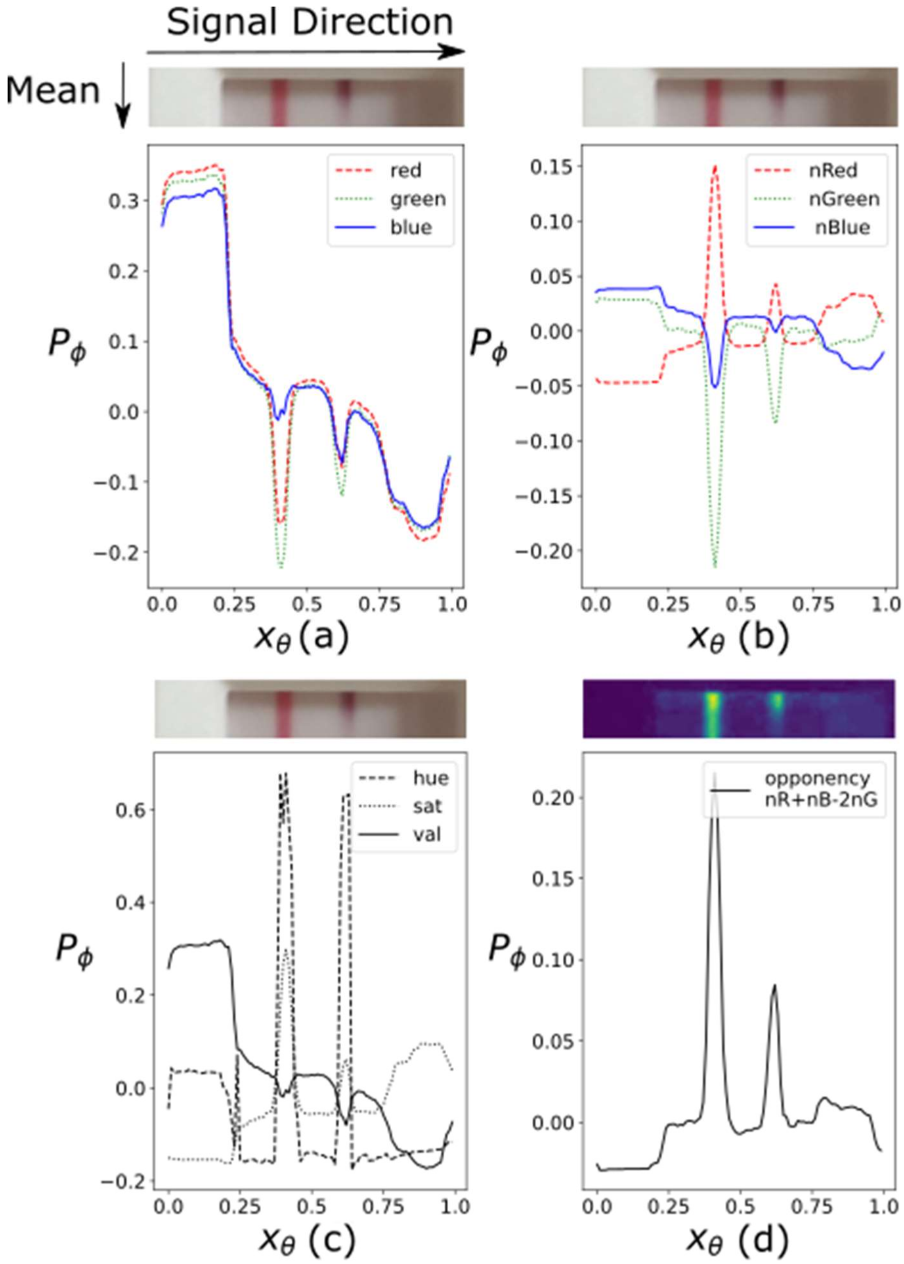

Figure S4: Normalised RGB (Red, Green, Blue) projection signatures for an invalid and valid Lateral Flow Immunoassay (LFIA). The figure shows examples of invalid and valid LFIA test result windows and their respective nRGB projection signatures, $P_{(R|G|B)}(x_\theta)$. Normalised Red (nRed) and Normalised Blue (nBlue) channels provide clear indicators of test status. We developed a simple algorithm that takes these signatures as input to determine the validity of the LFIA. The signatures also form the basis of semi-quantitative analyses presented later in this report. **a** Invalid test. **b** Valid test. Abbreviations are defined as follows: nGreen (Normalised green).

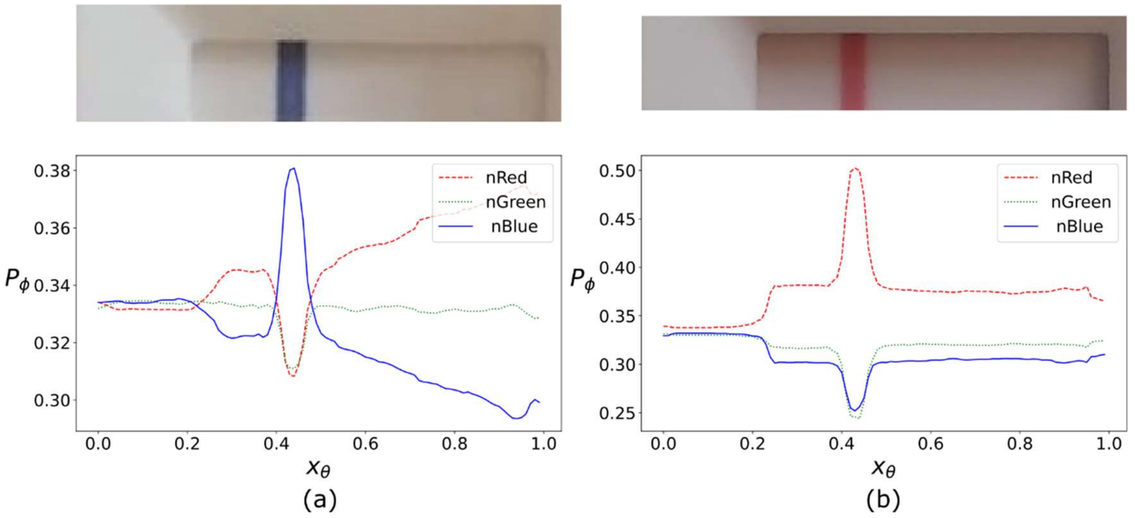

Figure S5: Opponency and edge-intensity projection signatures for seropositive and seronegative LFIAs. Simple edge detection can be used to localise areas corresponding to lines in the read-out window. Hand engineered algorithms are applied to bootstrap the labelling process, prioritising images for expert review and labelling when discrepancies between participant and algorithm are identified. This "targeted" sampling allows balanced datasets to be rapidly acquired, which was particularly important in the earlier period of the REACT-2 study. The figure shows examples of seropositive and seronegative Lateral Flow Immunoassay (LFIA) test result windows and their respective opponency and edge-intensity projection signatures. The algorithm labelled Heuristic-C in Tables S6 and S7 takes these signatures as input to determine the Immunoglobin G (IgG) result of the LFIA. **a** Seropositive result. **b** Seronegative result.

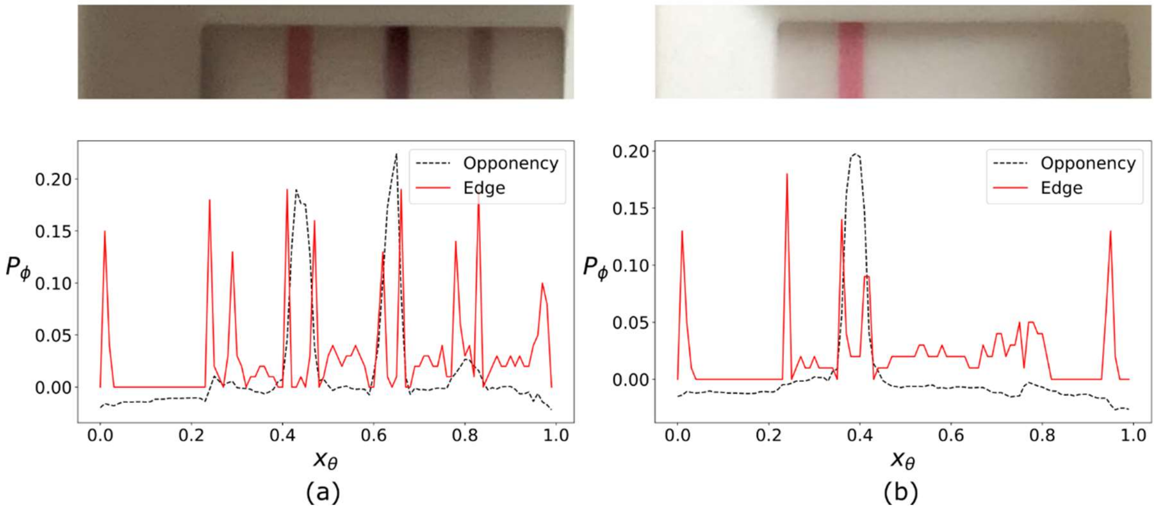

Table S1: Summary of REACT-2 data collected

Round	Period	No. of participants	No. of images	% of participants who supplied images	No. of images analysed	% of images analysed
Round-1 (R1)	20/06/2020 - 10/07/2020	109,075	94,700	86.62	93,252	98.47
Round-2 (R2)	30/07/2020 - 12/08/2020	111,057	96,817	87.18	95,508	98.65
Round-2B (R2B)	19/08/2020 - 31/08/2020	11,517	9,702	84.24	9,500	97.92
Round-3 (R3)	15/09/2020 - 27/09/2020	166,681	125,499	75.29	123,614	98.50
Round-4 (R4)	27/10/2020 - 10/11/2020	169,927	135,594	79.80	133,225	98.25
Round-5 (R5)	25/01/2021 - 08/02/2021	172,099	142,701	82.92	140,240	98.28
Total	-	740,356	605,013	81.72	595,339	98.40

Footnote: The difference between images available and images analysed is due to filtering in the pre-processing step (step 1) of the ALFA pipeline based on failed segmentation (pipeline could not identify the LFIA cassette or regions of interest in the image) or image corruption/error.

Table S2: Geometric priors used to filter out-of-distribution images from good quality images of Fortress COVID-19 LFIA. The geometric priors are formed from ratios of properties outlined in the table below. Optimisation of these pass ranges has yet to be conducted.

Geometric-priors	Property	Pass range
Result window / Blood well	Long-length	0.45 < r < 0.85
Result window / Blood well	Short-length	0.75 < r < 1.2
Blood well / LFIA (device)	Short-length	0.3 < 0.65
Result window / LFIA (device)	Short-length	0.3 < r < 0.65
Blood well / Result window	Area	0.25 < r
Blood well / Result window	Perimeter	0.55 < r

Table S3: Sociodemographic characteristics of the REACT-2 study (Rounds 1 to 5) participants, and for REACT-2 study participants for which (1) a photo was uploaded, (2) segmentation of the image was successful, and (3) a valid result was available.

Variable	Category	REACT-2 Cohort (Round 1 to 5)*	Photo uploaded Cohort*	Segmentation Successful Cohort*	Valid Result Cohort*
	All participants (N)	728,834	592,565	446,217	438,025
Sex	Female	408.575 (56.1)	331,491 (55.9)	250,145 (56.1)	245,604 (56.1)
	Male	320,259 (43.9)	261,069 (44.1)	196,070 (43.9)	192,419 (43.9)
Age group	18-24	42,598 (5.8)	37,804 (6.4)	28,927 (6.5)	28,219 (6.4)
	25-34	93,957 (12.9)	83,900 (14.2)	63,561 (14.2)	62,255 (14.2)
	35-44	121,351 (16.7)	107,255 (18.1)	81,234 (18.2)	79,838 (18.2)
	45-54	148,060 (20.3)	126,981 (21.4)	95,719 (21.5)	94,259 (21.5)
	55-64	150,491 (20.7)	122,052 (20.6)	91,269 (20.5)	89,611 (20.5)
	65-74	121,134 (16.6)	85,637 (14.5)	63,292 (14.2)	62,056 (14.2)
	74+	51,249 (7.0)	28,936 (4.9)	22,215 (5.0)	21,787 (5.0)
Ethnicity	Asian	25,998 (3.6)	21,184 (3.6)	16,575 (3.7)	16,310 (3.8)
	Black	6,142 (0.85)	4,931 (0.84)	3,787 (0.85)	3,696 (0.85)
	Mixed	8,983 (1.2)	7,703 (1.3)	5,867 (1.3)	5,743 (1.3)
	Other	6,331 (0.87)	5,095 (0.87)	3,977 (0.90)	3,907 (0.90)
	White	676,393 (93.4)	549,956 (93.4)	413,287 (93.2)	405,676 (93.2)
Education	No qualification	66,525 (9.2)	(7.5)	34,063 (7.7)	33,358 (7.7)
	Other	91,121 (12.6)	69,084 (11.7)	52,093 (11.7)	51,119 (11.7)
	GSCE	114,960 (15.9)	91,672 (15.6)	68,848 (15.5)	67,578 (15.5)
	Post-GCSE	200,687 (27.7)	168,284 (28.6)	126,397 (28.5)	124,078 (28.5)
	Degree or above	250,509 (34.6)	215,717 (36.6)	162,208 (36.6)	159,327 (36.6)

Footnote: *Round 2B participants were excluded as they are not part of the main REACT-2 study cohort.

Table S4: Sociodemographic characteristics associated with (1) photo upload, (2) successful segmentation of the image, and (3) a valid test result.

**Category**	**Photo uploaded ***			**Segmentation Successful ***			**Valid Result***		
All participants<br>Yes<br>No	728,834<br>592,565 (81.3%)<br>136,275 (18.7%)			592,565<br>446,217 (75.3%)<br>146,348 (24.7%)			446,217<br>438,025 (98.2%)<br>8,192 (1.8%)		
	Yes - n (%)	Unadjusted RR (95% CI)	^Adjusted RR (95% CI)	N (%)	Unadjusted RR (95% CI)	^Adjusted RR (95% CI)	N (%)	Unadjusted RR (95% CI)	^Adjusted RR (95% CI)
**Sex**									
Male	261,069 (81.5)	Ref	Ref	196,070 (75.1)	Ref	Ref	192,419 (98.1)	Ref	Ref
Female	331,491 (81.1)	0.995 (0.993-0.997)***	0.991 (0.989-0.993)***	250,145 (75.5)	1.005 (1.002-1.008)**	1.004 (1.001-1.007)*	245,604 (98.2)	1.001 (0.999-1.002)	1.001 (1.000-1.002)*
**Age group**									
18-24	37,804 (88.8)	Ref	Ref	28,927 (76.5)	Ref	Ref	28,219 (97.6)	Ref	Ref
25-34	83,900 (89.3)	1.01 (1.002-1.010)**	1.00 (0.998-1.007)	63,561 (75.8)	0.990 (0.983-0.997)**	0.990 (0.983-0.997)**	62,255 (98.0)	1.005 (1.003-1.007)***	1.005 (1.003-1.007)***
35-44	107,255 (88.4)	0.996 (0.992-0.999)*	0.997 (0.993-1.000)	81,234 (75.7)	0.990 (0.983-0.996)**	0.989 (0.983-0.996)**	79,838 (98.3)	1.009 (1.007-1.011)***	1.009 (1.007-1.011)***
45-54	126,981 (85.8)	0.966 (0.963-0.970)***	0.973 (0.969-0.977)***	95,719 (75.4)	0.985 (0.979-0.991)***	0.984 (0.978-0.991)***	94,259 (98.5)	1.012 (1.010-1.014)***	1.012 (1.010-1.014)***
55-64	122,052 (81.1)	0.914 (0.910-0.918)***	0.928 (0.924-0.932)***	91,269 (74.8)	0.977 (0.971-0.984)***	0.976 (0.970-0.982)***	89,611 (98.2)	1.008 (1.006-1.010)***	1.008 (1.006-1.010)***
65-74	85,637 (70.7)	0.797 (0.793-0.801)***	0.820 (0.816-0.825)***	63,292 (73.9)	0.966 (0.959-0.973)***	0.962 (0.955-0.969)***	62,056 (98.1)	1.006 (1.004-1.008)***	1.007 (1.005-1.009)***
74+	28,936 (56.5)	0.636 (0.631-0.642)***	0.661 (0.655-0.667)***	22,215 (76.8)	1.003 (0.995-1.012)	0.997 (0.988-1.005)	21,787 (98.1)	1.007 (1.004-1.009)***	1.008 (1.005-1.010)***
**Ethnicity**									
White	549,956 (81.3)	Ref	Ref	413,287 (75.2)	Ref	Ref	405,676 (98.2)	Ref	Ref
Asian	21,184 (81.5)	1.00 (0.996-1.008)	0.942 (0.937-0.948)***	16,575 (78.2)	1.04 (1.034-	1.04 (1.03-1.05)***	16,310 (98.4)	1.003 (1.001-1.005)**	1.003 (1.001-1.005)**

					1.049)***				
Black	4,931 (80.3)	0.987 (0.975-0.999)*	0.938 (0.927-0.949)***	3,787 (76.8)	1.02 (1.006-1.038)**	1.02 (1.01-1.04)**	3,696 (97.6)	0.993 (0.988-0.998)**	0.992 (0.988-0.997)**
Mixed	7,703 (85.8)	1.05 (1.046-1.064)***	0.985 (0.977-0.993)***	5,867 (76.2)	1.01 (1.000-1.026)*	1.01 (1.00-1.02)	5,743 (97.9(	0.997 (0.993-1.000)	0.997 (0.993-1.001)
Other	5,095 (80.5)	0.990 (0.978-1.002)	0.949 (0.938-0.960)***	3,977 (78.1)	1.04 (1.024-1.054)***	1.04 (1.02-1.05)***	3,907 (98.2)	1.00 (0.997-1.005)	1.001 (0.996-1.004)
**Education**									
Degree or above	215,717 (86.1)	Ref	Ref	162,208 (75.2)	Ref	Ref	159,327 (98.2)	Ref	Ref
Post-GCSE	168,284 (83.9)	0.974 (0.971-0.976)***	0.979 (0.976-0.981)***	126,397 (75.1)	0.999 (0.995-1.003)	1.001 (0.997-1.004)	124,078 (98.2)	0.999 (0.998-1.00)	1.000 (0.999-1.001)
GSCE	91,672 (79.7)	0.926 (0.923-0.929)***	0.950 (0.948-0.953)***	68,848 (75.1)	0.999 (0.994-1.003)	1.003 (0.999-1.008)	67,578 (98.2)	0.999 (0.998-1.00)	0.999 (0.998-1.000)
Other	69,084 (75.8)	0.880 (0.877-0.884)***	0.934 (0.931-0.938)***	52,093 (75.4)	1.003 (0.998-1.008)	1.009 (1.004-1.015)***	51,119 (98.1)	0.999 (0.997-1.00)	0.998 (0.997-0.999)*
No qualification	44,300 (66.6)	0.773 (0.769-0.778)***	0.873 (0.869-0.877)***	34,063 (76.9)	1.023 (1.017-1.028)***	1.03 (1.02-1.04)***	33,358 (97.9)	0.996 (0.995-0.998)***	0.996 (0.995-0.998)***

Footnote: +Round 2B participants were excluded as they are not part of the main REACT2 study cohort. RR Relative Risk; 95% CI 95% Confidence Intervals; *p<0.05, **p<0.01**, ***p<0.001; ^mutually adjusted for sex, age, ethnicity and education.

Supplementary Methods

Data acquisition

The immunoassay device used in these studies is the Fortress Diagnostics COVID-19 Total Antibody test from Fortress Diagnostics. Devices varied slightly in physical construction and visual appearance, requiring some care in either designing or detecting algorithms. Participants of the REACT-2 study were selected as described in earlier work[1]. Data were collected by IPSOS MORI, including self-readings and images uploaded from participants' devices. Images were pre-processed to remove geolocation or device tags, and then transferred to Imperial College London. A summary of the data collected and transferred is shown in Table S1.

Participants were provided with guidance on how to take suitable photographs. These instructions were refined over rounds, but the changes affected only the quantity of images that passed quality checks in the ALFA pipeline, as described below. The quality checks remove out-of-distribution data:

data that is significantly different from that used for algorithm training, avoiding unpredictable behaviour in subsequent ("downstream") hand-engineered algorithms or data-driven AI[2].

Pre-processing

The first stage of pre-processing is image segmentation. A 2D CNN identifies candidate regions of interest (ROI) for i) the LFIA itself, ii) the test result (read-out) window, and iii) the blood sample well. The segmentation output is then subjected to checks for segmentation failures using the geometric priors detailed in Table S2, they are rejected and flagged. Notable causes of such failures include i) submission of an image with no device visible (possibly an incorrect upload); ii) the test device being too small, or too blurred. Fewer than 2% of images are rejected in this way.

The centroids of the blood well and read-out window (depicted in Figure S2) were used to determine an approximate in-plane rotation for the device and used to rotate all read-out windows such that the long axis lay horizontally (i.e. to match the orientation depicted in Figure S3). The robustness of this method is due to the ROIs being relatively large and small errors in segmentation will not significantly affect the calculation of the centroid.

The region corresponding to the read-out window was cropped out to reduce the time for further processing and resized to *M*x100 - where *M* is variable to maintain isotropic pixels prior to producing projection signals (see below). The read-out window is separately resized to 50x100 during pre-processing before presenting to a 2D CNN classifier for the interpretation of the test result, assuming the test is valid (i.e. a red control line is detectable).

Heuristic Read-Out Algorithms and CNNs

The reading of the test result status – whether the test had been conducted correctly or not – was done using a heuristic algorithm making use of 3 separate colour spaces (detailed in S3), simple edge detection and the comparison of projection signals as a function of the long axis. The projection signals were calculated using Eq. S3-2 of Supplementary Notes 3. Examples of the projection signatures can be seen in Figure S3.

We used a peak detection algorithm from *scikit-learn* - `find_peaks()`, combined with a comparison of intensities along normalised red *nR* and normalised blue channels *nB* (Examples in Figure S4) to determine the status of the control line ("Valid" or "Invalid"). Images for which no test line could be detected were removed (less than 0.5%) from further processing. Detection of the status of the IgG line was done in stages: Phase 1, a hand-engineered algorithm applied to the opponency projection signal and an edge intensity signature (Examples shown in Figure S5), which was generated using Canny edge detection on the read-out window; Phase 2, a 1D CNN applied to the projection signals ((*R*,*G*,*B*), (*nR*,*nG*,*nB*), (*H*,*S*,*V*) and opponency (*O*)); Phase 3, a 2D CNN applied to the entire, rescaled read-out window. Discrepancies between user-reported results and Phase 1 and Phase 2 pipeline results were used to prioritise the review and labelling of images to locate "difficult" cases, effectively biasing the training, test and validation datasets used for training the final 2D-CNN readout networks in Phase 3. Ground truth for the read-out status was obtained by having a team of 6 trained observers review images. Since weakly positive cases are rare, the training dataset evolved over 8 months, and particularly improved – during Phase 3 – with the inclusion of weaker cases of immunity from vaccinated individuals.

Supplementary Discussion

Sociodemographic characteristics of REACT-2 Study-5

Table S3 summarises the sociodemographic characteristics of the REACT-2 study (Rounds 1 to 5) participants, and those REACT-2 study participants for which (1) a photo was uploaded, (2) segmentation of the image was successful, and (3) a valid result was available. Broadly, the sample profile of REACT-2 study participants who uploaded a photo of their test result was in keeping with that of the overall REACT-2 study cohort. Small differences were observed by age and education level. Older age groups (over 65s) and those with no qualifications were slightly underrepresented. This is consistent with findings exploring sociodemographic characteristics associated with the UK's digital divide and smartphone use[3,4]. The sample profiles of participants with image segmentation success and valid test result were representative of those who had uploaded a photo.

Table S4 summarises the findings of an exploratory analysis we performed using log-binomial regression to explore predictors of photo upload, segmentation success and valid test result by gender, age, ethnicity and education level. Photo upload, segmentation failure and an invalid result were associated, in relative terms, with all four predictors examined. However, in absolute terms, for segmentation failure and an invalid result, all percentage differences were very small. Photo upload was lower in females, older age groups, individuals from black, Asian and minority ethnic (BAME) groups, and individuals with fewer qualifications. Segmentation failure was marginally lower for females, 18-24-year-olds and 75+-year-olds, individuals from BAME groups, and individuals with fewer qualifications. Obtaining an invalid result was marginally lower for females, older age groups, individuals from Asian (and higher for Black) ethnic groups, and individuals with higher qualifications.

In summary, the sample profiles of the images analysed in this study were broadly in keeping with the profile of the overall REACT-2 study cohort, and absolute differences in gender, age, ethnicity and education level were small, despite being predictors of photo upload, segmentation failure and an invalid result. Therefore, we would not expect estimates of antibody prevalence based on automated analysis to be significantly bias. However, consideration should be given to increasing photo upload in those population subgroups in which internet access and smartphone use are known to be lower. This could include giving these participants the option of an alternative image capture approach, for example, a trained study staff member talking through the image capture process over the phone with the participant in real-time or coming to the participants home at the time of the test to take the photo.

Supplementary Notes 2

Table S5: The 1D CNN architecture. "conv" is 1D convolution (no. of inputs, no. of outputs, filter length), "FC" (No. of inputs, no. of outputs), "batchnorm" are 1D batch normalisations and "MaxPool" are 1D max pooling functions with kernel size of 2. Note that "Input(10)" refers to 10 projection signatures of length 100, please look at Figure S3.

Model architecture
Input (10)
Conv(10,30,5)
Batchnorm + MaxPool
Conv(30,30,5)
Batchnorm + MaxPool
Conv(30,20,5)
Batchnorm + MaxPool
FC(180,170)
FC(170,70)
FC(70,1)
Sigmoid

Supplementary Methods

Segmentation

A deep Convolutional Neural Network (CNN), based on a U-Net architecture[4], was trained to identify candidate regions of interest (ROI). Training of this network, known as *dhSegment*[5], involves using pre-trained weights for the "encoding part" of the network and fine-tuning the "expansive part" of the network (which is mapping the encoded feature maps to full resolution feature maps). The raw output undergoes post-processing consisting of connected component analysis and small region removal, providing a simplified output segmentation. The network is implemented in TensorFlow.

We randomly selected an initial 498 LFIA images for developing the segmentation CNN (Originally 500, however there were file format issues with two images). Regions were manually labelled using VGG Image Annotator (VIA)[6] by 2 people, providing a representative sample for training and testing. We split the dataset into fixed train, test, and validation sets: 373 for training, 42 for validation and 83 for testing. Dice scores were found to be: 0.973 for the LFIA cassette, 0.943 for the read-out window, and 0.921 for the blood well.

Classification ground truth

Ground truth for the read-out status was obtained by having a team of 6 trained observers review images. Since weakly positive cases are rare, the training dataset evolved over 8 months, and particularly improved – during Phase 3 – with the inclusion of weaker cases of immunity from vaccinated individuals (Main manuscript, Table 2). This was important as the weakly positive cases were identified as a source of false negative readings by participants of the REACT 2 study when assessed by human experts.

Classification 1D CNN (Phase 2 Read-out Training)

A popular deep neural network architecture for image classification is the 2D CNN. However, as shown in Supplementary Notes 1 (Figure S3), the LFIA read-out window has a strong linear structure that can be collapsed into 1D signatures. 1D signatures are easily human interpretable, providing semi-quantitative amplitudes, which will be used in subsequent work. Peak detection algorithms can also be applied to detect locations corresponding to lines representing control and readout, with minimal data. 1D CNNs typically consume far less data for training than 2D CNNs, making them suitable to bootstrap the labelling process, and particularly for finding rare but important cases to balance larger training sets to support more sophisticated architectures. We used the ten signatures (Supplementary Notes 1, Figure S3) to explore five different candidate 1D CNNs (implemented in Pytorch) varying in the number of convolutional filters, filter lengths and number of layers. The final 1D CNN architecture selected for read-out interpretation is shown in Table S5.

Classification 2D CNN (Phase 3)

A second 2D CNN (implemented in Tensorflow) is trained for accurate read-out interpretation and yielded the best sensitivity. This CNN utilises a MobileNetV2 architecture with pretrained weights learned on the ImageNet[8] dataset. The final 2D network is based on previous work by the McKendry group and the i-sense interdisciplinary research collaboration (IRC) (www.i-sense.org.uk) at University College London; the main paper describes performance in two separate experiments (CE1 and CE2) which tradeoff slightly the specificity and sensitivity of readout interpretation.

The 2D CNNs for segmentation and classification, as well as the (more recent) transformer network, were trained and fine-tuned on an RTX6000 with 24GB of memory. Training time is usually no more than half a day for any one network.

Supplementary Notes 3

Table S6 Classification Experiment 1: A comparison of the performance of the projection-based peak detection algorithm described in this Supplementary material. 1D CNNs make use of the projection colour spaces we describe in the Supplementary Methods of these notes., whilst "Heuristic C" represent the best parameter selection for peak detection. Bear in mind that all of these techniques for pre-processing of the data can be viewed as a combination of i) dimensionality reduction and ii) KL projection, effectively reflecting either geometric or statistical algorithms. Whilst these algorithms work very well for determining the status of the control line (see the Supplementary Methods of these notes), the same principle displays relatively poor sensitivity, and this is due to the presence of weak positives. In contrast, the 2D CNN model displays the best combination of specificity and sensitivity.

Model/Heurisitic/Participants	Specificity	Sensitivity	Accuracy	Cohen's Kappa
2D CNN	0.994	0.971	0.983	0.966
1D CNN Model 1	0.995	0.831	0.917	0.832
1D CNN Model 2	0.968	0.853	0.913	0.824
1D CNN Model 3	0.999	0.879	0.941	0.882
1D CNN Model 4	0.988	0.833	0.914	0.827
1D CNN Model 5	0.990	0.900	0.946	0.892
Heuristic-C	0.994	0.757	0.881	0.759
Study Participants	0.961	1	0.980	0.959

Table S7 Classification Experiments when substantial weak positives are included. This provides a comparison between carefully tuned peak-detection algorithm and CNN performance for data containing a significant proportion of weak positive samples. Thus, the notion of sensitivity and specificity, though probabilistically well defined, are only reliable when the dataset correctly reflect the background priors of weak cases amongst positive cases. This is a critical point in representing the sensitivity of detection, and is the key reason that the more sophisticated 2D CNN is required.

Model/Heurisitic/Participants	Specificity	Sensitivity	Accuracy	Cohen's Kappa
2D CNN	1	0.852	0.949	0.883
2D CNN (CE2, retrained)	0.987	0.901	0.958	0.905
1D CNN Model 1	0.969	0.825	0.920	0.701
1D CNN Model 2	0.965	0.810	0.912	0.667
1D CNN Model 3	0.968	0.844	0.926	0.733
1D CNN Model 4	0.941	0.854	0.911	0.678
1D CNN Model 5	0.960	0.852	0.923	0.726
Heuristic-C	0.976	0.487	0.815	0.525
Study Participants	0.974	0.679	0.873	0.699

Supplementary Methods

Classical algorithms

The quasi-linear structure of the LFIA window does indeed suggest that the application of relatively simple operations e.g. linear projections – similar to principal components analysis – to solve the problem of detecting the location and status of control lines. Indeed, we use standard algorithms of a mature Python library (scipy-signal) to detect these peaks. However, we also make use of a colour transformation that uses non-orthogonal bases of colour space that are *very* similar to a Karhunen-Loeve transform on 3D colour space to increase the detectability of the control line using these algorithms. Whilst these are quite adequate for the *control* line (with a small number of exceptions), straightforward 1D statistical peak detection is inappropriate for the IgG status line due to two key factors:

i) The presence of weak positives, which reduce contrast to below the typical noise threshold using linear projections.

ii) The presence of blood leakage into the window which – though providing high degrees of specificity, does so with reduced sensitivity, bringing the

Indeed, we apply such a technique to peak detection for the **control line status**, and this works quite robustly.

In the following sections of this Appendix to our response to Reviewers' Comments, we present the evidence that – although a simpler approach to status detection seems feasible – it does not match the individual precision and sensitivity that is attained by the 2D CNN.

The reason for this is relatively intuitive (in hindsight!): although the ideal image sample contains blood only in the well, either leakage or "spatter" will sometimes lead to blood that falls into the readout window (this is a more common occurrence than one would anticipate). Damaged devices can also display significant departures from the expected appearance. The presence of such cases is not always discernible in 1D projection space from a positive IgG result: it is best captured from the segmented window of the original 2D image.

Read-out Algorithms (interpreting the result, post segmentation)

Having substantially reduced the size and complexity of the image data through the pre-processing steps, we are in a position to focus on the read-out window. Due to mapping the pixel data of the read-out window into a reference axis (x_θ, y_θ), we note that the image has a strongly linear structure. The alignment of this linear structure with the representation axis (i.e. row-column organisation of the image) means that we can apply low-complexity algorithms to perform several of the steps needed for interpreting read-out, without recourse to data-driven training.

The read-out window of the Fortress device provides a control line that switches colour from blue to red when the test has been correctly performed. Thus, a key stage that gates subsequent interpretation is the presence of the control line and the reading of its colour.**Colour Spaces**

The raw RGB values of the pixel intensities corresponding to the read-out window can shift with changes in lighting, the colour calibration of the device and the presence of shadows. In practice,

colour-space transformations to the raw pixel values can be a convenient way of obtaining at least partial invariance to nuisance sources of colour shift. We found it convenient to use three different colour transformations, depending on the need to be colour-selective or approximately colour-invariant.

We use ten colour channels across four colour spaces. These are (R, G, B) (original pixel values), (nR, nG, nB) (normalised colour spaces), where $nR = R/(R + G + B)$ etc, (H, S, V) and a single-channel opponent colour space, $O = nR - 2nG + nB$. Like the (H, S, V) colour space, opponent colour spaces correlate well with perceptual notions of colour. The O channel, in particular, is recognisable as proportional to one channel of an Ohta (Tkalcic et al.[9]) colour space, which is an approximation to one channel of the Karhunen-Loeve (KL) decomposition of colours for natural imagery. The Ohta space is known to be a good choice for colour image segmentation (Kartikeyan et al.[10]) and visual descriptors (Payne et al.[11]).

Projection Signatures

Rather than using principal components analysis in colour space, we simply project (sum, or take an average) of the intensity data along the y_θ direction, yielding a function of x_θ. These projection signatures, $P_\Phi(x_\theta)$ are produced for each channel of each of three selected colour spaces. Specifically, for any colour-spatial field in the aligned coordinate system $f_\Phi(x_\theta, y_\theta)$, for $\Phi \in \{R, G, B, nR, nG, nB, H, S, V, O\}$ we approximate the 2D to 1D projection operator:

$$P_\Phi(x_\theta) = K \int f_\Phi\,(x_\theta, y_\theta) dy_\theta \qquad \text{Eq. S3} - 1$$

where K is a normalising constant that compensates for different zoom factors. In practice, the integral is simply approximated by taking an average over the rows (or columns) of the two-dimensional image array representing the normalised image window corresponding to the read-out:

$$P_\Phi(n) = \frac{1}{M} \sum_{m=1}^{M} f_\Phi(m, n) \qquad \text{Eq. S3} - 2$$

where Φ is selected from each of {*R, G, B, nR, nG, nB, H, S, V, O*}; the first 9 of these channels are taken from RGB, normalised RGB (trichromatic coefficients) and HSV colour space. The final channel, *O*, is an opponency colour space defined by:

$$O = nR - 2nG + nB$$

Illustrations of the projection signatures are provided in Figure S3. Due to the white background of the test read-out window, the presence of red or blue lines is somewhat counterintuitive in the native (R, G, B) space, manifesting as dips in intensity on the primary colour channels. The normalised channels provide a slightly more obvious depiction, and we can observe the differences in appearance between the control lines for these cases (see also Figure S4).

Detection of the Control Line

The first step to interpret the LFIA result is to locate the control line. The pipeline uses the opponency signature (Eq S3-1) and runs a peak detection heuristic algorithm (*scipy-signal* module,

find_peaks) to find a peak corresponding to the control line. Because of the rescaling operation to a reference coordinate system (using segmentation and image rescaling), performing peak detection on this approach works to a satisfactory level.

This algorithm takes 5 parameters of height, width, relative height, distance, and prominence. The optimal settings for these five parameters was set by a grid search across three hyper-parameters $\alpha_h \in [0.9,1.1]$, $\alpha_w \in [0.1,0.5]$ and $\alpha_p \in [0.005,0.016]$, then setting the *height* parameter of `find_peaks()` to $\mu \times \alpha_h$, *prominence* to $\mu \times \alpha_p$ the *width* parameter to $100 \times \alpha_w$; μ is the average amplitude of the projection signal. The parameter *relative_height* was fixed at 0.5, and *distance* was fixed at 100×0.1.

If this simple approach fails to locate the control line, then the LFIA result is deemed 'unreadable' and removed from the pipeline.

Reading the control line status

A 2nd heuristic algorithm determines the validity by reviewing the normalised red (nR) and blue (nB) values at the control-peak location. For invalid tests, the nB value is higher than nR. The relationship is consistently reversed for valid tests, and Figure S4 illustrates this. Invalid LFIAs are removed from the pipeline and reported as invalid test results, whilst the images deemed to be valid; move to the later stages of analysis.

In testing, this second heuristic algorithm on dataset 2, all examples are classified correctly for test *validity*. Though dataset 2 is small, and the results may not reflect the performance in the field, we can assume that the errors of Heuristic-B will be few. For future work, we can collect the data that participants have labelled invalid and create ground truth, a more trivial task than labelling the IgG status of the LFIA. Should this approach begin to fail significantly, a 1D or 2D CNN could be developed to classify validity.

Seroprevalence Read-out: IgG Status

Initially, with the limited amount of labelled data, we developed an algorithm based on established peak-detection techniques to classify the IgG Status of samples. As the amount of data we labelled increased, more data-intensive methods replaced this approach, leading to the creation of first 1D and then 2D CNNs. The initial development and testing, Classification Experiment 1 (CE1), used Data set 3 The development set consists of samples from REACT-2 Study-4 and Study-5's R1. The test set came from Study-5's R2, removing any risk of data leakage; additionally, Study-5's R2 contained participant response data for comparison against both human experts and algorithm readings. For CE1, we did five-fold cross-validation on just the development set, ensuring that it was suitable for training; we then retrain on the whole development set and report the testing of the final model on the test set. Reported metrics are specificity, sensitivity, overall accuracy and Cohen's kappa.

Taking advantage, again, of the linear structure, we designed an initial data bootstrapping algorithm using the opponency signature and an edge intensity signature, which is only used for IgG status classification. The principles for detecting the control line are also applied; the heuristic looks for distinct peaks at identified locations. For a seropositive image, we require: a peak in opponency space, and to differentiate from possible blood leakage, two peaks in the edge-intensity space. The signatures are shown in Figure S5.

Using data-driven deep learning to finesse IgG status detection

The performance of the algorithms based on the colour space and geometric projection was excellent for strong (high visual contrast) IgG seroprevalence, but inadequate for weak IgG line. Weak IgG lines can be caused by a variety of factors, but the most likely cause is due to waning or weak immune response.

We found that the detection of weak lines using peak detection to be below that of expert readers, and in some cases, even of members of the public. Thus, we explored data driven machine learning with incrementing degrees of complexity: low parameter-count networks using 1D CNNs, then high-parameter count networks and transfer learning using 2D CNNs.

1D CNN with projection signatures

A popular deep-learning method is the 2D CNN; however, the LFIA read-out has a strong linear structure that facilitates collapsing the colour information into 1D signatures. We, used ten signatures from different colour spaces, examples in Figure S3, as inputs to our 1D CNNs. Five 1D network architectures were used, varying in the number of convolutional filters, the filter lengths and the number of layers. These variations enabled us to explore how architecture parameters might affect the performance. The 1D CNNs were implemented with PyTorch (Paszke et al.[12]), and training was completed over 100 epochs, using binary cross-entropy loss, and a learning rate of 0.0013. The development set was split into a 90:10 ratio for training: validation and batch sizes set to 2. Several random generator seeds were used, providing a mean and standard deviation (across seeds).

Alongside the networks, we developed custom data augmentation routines for the projection signatures. These included shifts in y_θ, scale changes and one-dimensional blur. These routines were selected as being appropriate augmentation routines for the one-dimensional signatures, reflecting the nature of the data transformations likely to be encountered.

2D CNN with normalised read-out images

As more data was accumulated, we were able to leverage discrepancies between user-submitted, peak detection algorithms, then 1D-CNNs to the point where sufficient data was available to allow training the 2D CNN, implemented in Tensorflow, for read-out interpretation. This CNN utilised a MobileNetV2 (Sandler et al.[13]) architecture with pretrained weights learnt on ImageNet (Deng et al.[8]). The CNN was fine-tuned using the development set, over 100 epochs using a sparse categorical cross-entropy loss function, with a learning rate set to 0.001. Reported performance for this network is obtained from the test-set.

Supplementary Discussion

Classification Experiment 1 Results

With regards to specificity, all methods perform better than the study participants. However, this is not the case for sensitivity where the participants are perfect, followed by the 2D CNN, 1D CNN models and finally, Heuristic-C. Looking at overall accuracy, the 2D CNN and participants perform similarly, both being better than the 1D CNNs and a heuristic approach based on colour-space projections and peak detection. For Cohen’s Kappa with the expert, we can see that the 2D CNN has

performed the best, with the value representing 'almost perfect agreement.' These results are promising as they initially imply that a 2D CNN, in general, could perform at a level on par with experts and hence auto-validation for LFIA could be possible. Although the 1D CNNs are not at the standard of the 2D CNN, the method still shows promise as it has high specificity while being nearly 27-50 times smaller than the 2D CNN, see Table S6.

Classification Experiment 2 Results

Based on the apparent high-quality of these results, we implemented the methods into ALFA and deployed it to analyse the 'wild' data consisting of 500,000 images. A pattern emerged, many discrepancies were flagged where participants reported seropositive, but ALFA reported seronegative. Experts reviewed a sample of the discrepancies, discovering cases of 'weak IgG positives.' Detection of these cases is the objective of Classification Experiment 2 (CE2, shown in Table S7), which utilises samples from R5 of Study-5.

Cases of the Weak Positives

As REACT-2 Study-5 was ongoing throughout the pandemic, R5 was the first round where a small sample of participants had received their 1st vaccination dose. It was expected that the participants who received their first dose at least 21 days prior to completing the LFIA; should be seropositive; however, some reported seronegative tests. An expert review of these cases was completed, confirming the presence of weak IgG positive examples in addition to creating more training data. This new data set was used for CE2, where the networks were retrained and tested using the new data sets. Most training parameters remained the same from CE1 to CE2; however, the number of epochs increased from 100 to 150, and the training and validation batch sizes increase to 10 and 5, respectively.

Table S7 shows the results of CE2 and confirms the necessity of providing difficult cases in the training data. As the 2D CNN model trained in CE1 was the best performing method with a near-perfect agreement with an expert, we applied it to the test set of CE2. The results show that CE1's 2D CNN could not handle the weak positives; however, after retraining the model (CE2, retrained), we see improvement implying a cycle of iterative improvement is possible. This cycle would involve applying the best performing method to the 'wild' data, reviewing the discrepancies, identifying weak cases and retraining the network. Still, ground truth labelling is a labour-intensive task and requires a certain level of expertise; a solution could be to open-source the data set by scrubbing personal information and providing the labelling methods (e.g. Oxford University's Visual Geometry Group (VGG) Image Annotator, VIA).

Another vital message is that although the weak positives have caused the performance of the methods to decrease compared to expert reviews, we see that all methods, including Heuristic-C, outperform the study participants. This suggests that once implemented into ALFA, the algorithms can identify reporting mistakes.

Supplementary Notes 4

Figure S6: Ensemble averages for different user-reported test-result-conditions. Abbreviations are defined as follows: IgG (Immunoglobin G), and IgM (Immunoglobin M). N denotes number of samples.

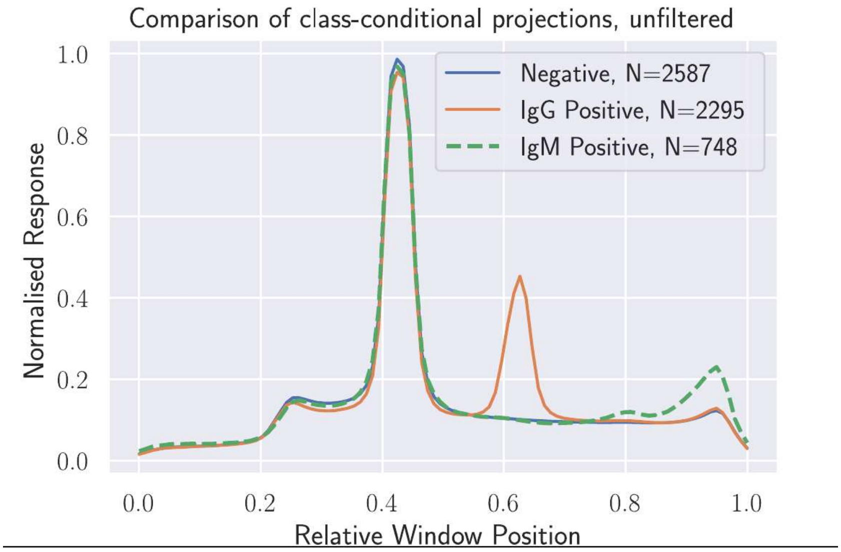

Figure S7: Examples of faulty devices and blood leakage. a broken devices**. b** “heavy” blood leakage. **c** “light” blood leakage. Abbreviations are defined as follows: nR (Normalised red), nB (Normalised blue), and nG (Normalised green).

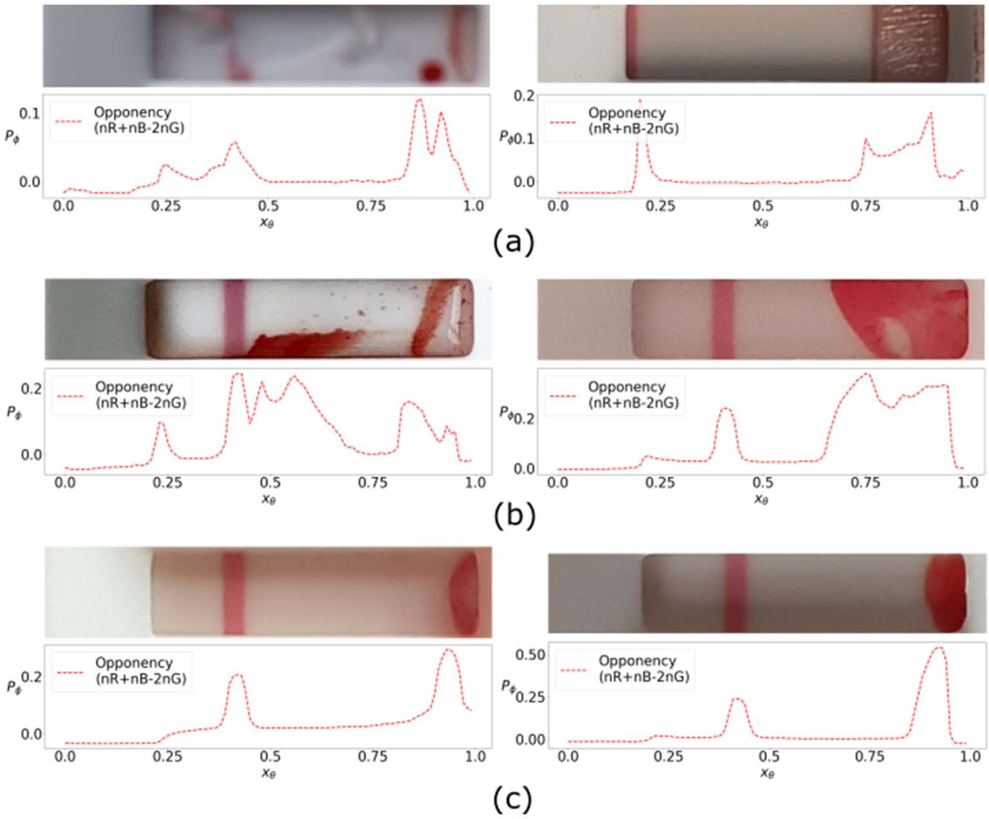

Figure S8: Difference between filtered and unfiltered Immunoglobin M (IgM, + denotes a positive results) ensemble signatures. N denotes the number of samples.

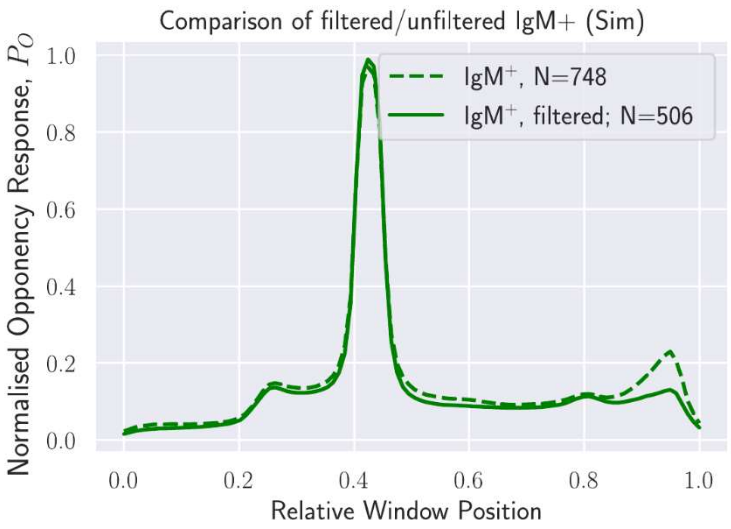

Supplementary Methods

Anomaly Detection

The opponency projection signature was found to be a good proxy for two-dimensional interpretation of the test window. Though it does not capture intensity variations that are parallel to the lines of the assay, it enables semi-quantitative analyses through alignment and ensemble-averaging of the signals. By grouping, aligning, and ensemble-averaging the projection signatures corresponding to different user-reported results, we observed an elevated region of the signature in users who self-reported as being IgM positive (Figure S5, green). This region was not at the expected location for the IgM line, but rather toward the edge of the read-out window. Closer scrutiny revealed that many self-reported IgM positive cases were due to leakage of the blood sample into the read-out window in such a way as to produce a clear (and thin) red line. Training a system to detect blood leakage – as well as broken devices – would be a natural next step, but variations in details of blood-leakage and spoiled tests vary dramatically and can be difficult to find in a large corpus of images. Observing the high degree of clustering of opponency signals around positive and negative cases (Figure S5, blue and orange), we used two simple measures for anomaly detection, both based on comparisons between a template signal and the fixed length (100 spatial samples) opponency signal obtained from the read-out window of a specific image, $P_O^{(i)}(n), n = 0,1,2,\dots 99$ (see Supplementary Information S3). The comparisons are based on normalised cross-correlation between the template signal and a candidate opponency signal:

$$\rho^{(i)}(k) = \langle\, \mu(n)/\|\mu(n)\|\,, P_O^{(i)}(n+k)\, / \left\| P_O^{(i)}(n) \right\| \,\rangle$$

Where $\langle a, b\rangle$ denotes the inner product between two arrays or signals, and $\|a\|$ denotes the 2-norm.

Anomalies are flagged under either of two conditions:

$$\arg\max[\rho^{(i)}(k)] > \eta/2$$

and

$$\max[\rho^{(i)}(k)] < \gamma$$

The template signal is defined by $\mu(n) = \mathbb{E}[P_O(n)]$, where the expectation, $\mathbb{E}$, is taken over a sufficiently large ensemble of projection signals drawn at random from all classes. We found little change in the shape of the template, $\mu(n)$, beyond a sample size of 5,000 signals. The value of η is set to be 1/3 of the length of the read-out window, since shifts of both IgG positive and negative projections were very unlikely to be outside of this range; the value of γ (a cosine similarity measure, $0 \leq |\gamma| \leq 1$) is close to 1 for most sample classes; we found (empirically) that setting a value of 0.85 provided a good filter for unusual projection signatures in a manner that was uncorrelated with expertly-determined IgG status.

Anomalies detected by either of these two conditions are flagged for expert human review; a sample of the read-out windows containing such cases is shown in Figure S6. After such anomalies are removed through filtering, the elevated section of the opponency signature – and therefore user-reported false positives – is significantly reduced, leaving us with a higher proportion of true, user-reported IgM positives (Figure S8).

Supplementary References

1. Ward, H. *et al*., (2021). "Prevalence of antibody positivity to SARS-CoV-2 following the first peak of infection in England: Serial cross-sectional studies of 365,000 adults". *The Lancet Regional Health Europe* **4,** 100098.

2. Ren, J. *et al*., (2019). "Likelihood Ratios for Out-of-Distribution Detection". In 33*rd Conference on Neural Information Processing Systems* (*NeurIPS 2019*), 32, Vancouver, Canada.

3. Office for National Statistics, (2019). "Exploring the UK's digital divide". [Available from: https://www.ons.gov.uk/peoplepopulationandcommunity/householdcharacteristics/homeinternetandsocialmediausage/articles/exploringtheuksdigitaldivide/2019-03-04].

4. OFCOM, (2021). "Adults' media use and attitudes report 2020/21" [Available from: https://www.ofcom.org.uk/research-and-data/media-literacy-research/adults/adults-media-use-and-attitudes].

5. Ronneberger, O. *et al*., (2015). "U-Net: Convolutional networks for biomedical image segmentation". *In International Conference on Medical Image Computing and Computer-Assisted Intervention*, pp234-241.

6. Oliveira, S.A. *et al*., (2018). "dhSegment: A generic deep-learning approach for document segmentation". In 2018 16*th International Conference on Frontiers in Handwriting Recognition* (*ICFHR*), pp7-12.

7. Dutta, A. and Zisserman, A., (2019). "The VIA annotation software for images, audio and video". In *Proceedings of the 27th ACM International Conference on Multimedia,* (2019), pp2276-2279. https://doi.org/10.1145/3343031.3350535.

8. Deng, J. *et al*., (2009). "Imagenet: A large-scale hierarchical image database". In 2009 *IEEE Conference on Computer Vision and Pattern Recognition*, pp248-255.

9. Tkalcic M. and Tasic, J. F. , (2003). “Colour spaces: perceptual, historical and applicational background”. *The IEEE Region 8 EUROCON 2003. Computer as a Tool,* Vol. 1 pp304-308. https://doi.org/10.1109/EURCON.2003.1248032.

10. Kartikeyan, B. *et al*., (1998). “A Segmentation Approach to Classification of Remote Sensing Imagery.*” International Journal of Remote Sensing* 19 (9), pp1695–1709.

11. Payne, A. and Singh, S., (2005). “A Benchmark for Indoor/Outdoor Scene Classification”. In *International Conference on Pattern Recognition and Image Analysis*, pp711–18.

12. Paszke, A., *et al*., (2019). “PyTorch: An imperative style, high-performance deep learning library”. In *Advances in Neural Information Processing Systems* (*NeurIPS 2019*), 32, pp8026-8037.

13. Sandler, M. *et al.*, (2018). “MobileNetV2: Inverted Residuals and Linear Bottlenecks.” In *IEEE/CVF Conference on Computer Vision and Pattern Recognition*, pp4510–4520. https://doi.org/10.1109/CVPR.2018.00474.
