# Supplementary material for: Machine learning to support visual auditing of home-based lateral flow immunoassay self-test results for SARS-CoV-2 antibodies
Source: Commun Med (Lond). 2022 Jul 6;2:78. doi: 10.1038/s43856-022-00146-z (PMC9259560; doi:10.1038/s43856-022-00146-z)
Supplement: Supplementary file 6 — Reporting Summary [file 43856_2022_146_MOESM6_ESM.pdf]

nature research

Corresponding author(s): Nathan C K Wong, Christina J Atchison, Rachel A McKendry, Anil A Bharath

Last updated by author(s): Jun 8, 2022

Reporting Summary

Nature Research wishes to improve the reproducibility of the work that we publish. This form provides structure for consistency and transparency in reporting. For further information on Nature Research policies, see our Editorial Policies and the Editorial Policy Checklist.

Statistics

For all statistical analyses, confirm that the following items are present in the figure legend, table legend, main text, or Methods section.

n/a	Confirmed	
☐	☒	The exact sample size (*n*) for each experimental group/condition, given as a discrete number and unit of measurement
☐	☒	A statement on whether measurements were taken from distinct samples or whether the same sample was measured repeatedly
☒	☐	The statistical test(s) used AND whether they are one- or two-sided<br>*Only common tests should be described solely by name; describe more complex techniques in the Methods section.*
☐	☒	A description of all covariates tested
☐	☒	A description of any assumptions or corrections, such as tests of normality and adjustment for multiple comparisons
☐	☒	A full description of the statistical parameters including central tendency (e.g. means) or other basic estimates (e.g. regression coefficient) AND variation (e.g. standard deviation) or associated estimates of uncertainty (e.g. confidence intervals)
☒	☐	For null hypothesis testing, the test statistic (e.g. *F*, *t*, *r*) with confidence intervals, effect sizes, degrees of freedom and *P* value noted<br>*Give P values as exact values whenever suitable.*
☒	☐	For Bayesian analysis, information on the choice of priors and Markov chain Monte Carlo settings
☒	☐	For hierarchical and complex designs, identification of the appropriate level for tests and full reporting of outcomes
☐	☒	Estimates of effect sizes (e.g. Cohen's *d*, Pearson's *r*), indicating how they were calculated

Our web collection on statistics for biologists contains articles on many of the points above.

Software and code

Policy information about availability of computer code

Data collection	No software or code was used for primary data collection from study participants of the REACT-2 programme.
Data analysis	The custom software (ALFA pipeline) was used to analyse the REACT-2 Study-5 Dataset. This pipeline will be made available on a community repository alongside a standalone demo (Currently this repo: https://github.com/TianhongDai/react2-code, is hosting the network to be carried forward in future work). The pipeline is written in python and utilises several libraries, key libraries include: Pytorch, tensorflow, opencv, pandas, and scikit-learn. Version specification for the libraries and python varied for different components in the pipeline and will be detailed in the installation files. The pipeline also makes use of the dhSegment framework (https://github.com/dhlab-epfl/dhSegment) and builds on the 2D CNNs produced in previous works by the McKendry group and i-sense interdisciplinary collaboration at University College London. Details of these are found in Supplementary Information 2 (Appendices S2-A and S2-D).

For manuscripts utilizing custom algorithms or software that are central to the research but not yet described in published literature, software must be made available to editors and reviewers. We strongly encourage code deposition in a community repository (e.g. GitHub). See the Nature Research guidelines for submitting code & software for further information.

Data

Policy information about availability of data

All manuscripts must include a data availability statement. This statement should provide the following information, where applicable:

- Accession codes, unique identifiers, or web links for publicly available datasets
- A list of figures that have associated raw data
- A description of any restrictions on data availability

Code and additional data to support the figures is freely available on this GitHub repo: https://github.com/TianhongDai/react2-code. The DOI is 10.5281/zenodo.6616921.

For the underlying data, access to this data is restricted due to ethical and security considerations. To obtain ethics approval from the South Central Berkshire B Research Ethics Committee (REC) and Health Regulator Authority (HRA), we agreed that we will preserve the confidentiality of participants taking part in the study and fulfil transparency requirements under the General Data Protection Regulation for health and care research. We also agreed that all REACT study data is to be held securely and processed in a Secure Enclave. This is an isolated environment within Imperial College for the processing of health-related personal data. It provides a framework that satisfies Information Governance requirements that come from several sources.

The Secure Enclaves are compliant with the requirements of major data providers (e.g. ONS, NHS Digital and NHS Trusts), as well as flexible to incorporate additional requirements a group may be subject to. The enclaves are ISO27001 certified.

These restrictions apply to all the study data, both qualitative and quantitative. We do not allow any line list data to be taken from the secure enclave because of the risk of cross-referencing and deductive disclosure. A researcher can request access to the data held in the Secure Enclave by emailing react.access@imperial.ac.uk. Access would be granted to researchers for the purposes of further research subject to approval by the data access committee and after signing a data access agreement to ensure no disclosure of potentially identifying details.

Source data for Figures 3a, b, and c is available in Supplementary Data 1, 2, and 3 respectively. Figure 3c utilises data from Supplementary Data 2 and 3.

Field-specific reporting

Please select the one below that is the best fit for your research. If you are not sure, read the appropriate sections before making your selection.

☒ Life sciences ☐ Behavioural & social sciences ☐ Ecological, evolutionary & environmental sciences

For a reference copy of the document with all sections, see nature.com/documents/nr-reporting-summary-flat.pdf

Life sciences study design

All studies must disclose on these points even when the disclosure is negative.

Sample size

No sample size calculation was performed for the study described in this manuscript. All images submitted by REACT-2 study participants were used.

Sample size with regard to the training and development of the ALFA pipeline was not calculated. As the REACT-2 Study-5 series of surveys was ongoing, the amount of data available was increasing. Additionally, the amount of data which could be labeled was dependent on the number of reviewers and their availability.

Data exclusions

Images could not be used if the LFIA device or test result window was not present in the image or due to image corruption/error.

Replication

The results of this manuscript are produced by passing all REACT-2 Study-5 images through the ALFA pipeline. Replication is possible by rerunning the analysis using the same models and scripts. The performance of the 1D CNN was replicated in a separate project which was reviewing the types of projection signatures supplied as input.

Randomization

REACT-2 is a series of non-overlapping cross-sectional population surveys of the prevalence of SARS-CoV-2 antibodies in the community in England, UK. REACT-2 rounds 1 to 5 were conducted at 1- to 2-month intervals between June 20th 2020 and February 8th 2021. At each round, we contacted a random sample of the population by sending a letter to named individuals aged 18 or over from the National Health Service (NHS) patient list (almost the whole population). We then sent respondents a test kit and instructions by post, as well as a link to an online instructional video. We asked participants to perform the test at home and complete a questionnaire, including reporting of their test result. They were also asked to submit a photograph of their completed test via an online portal using instructions and a template provided.

All images submitted by REACT-2 study participants were available for use in the study described in this manuscript. Subsets of images were used to train and improve the machine learning algorithms for high concordance with visual interpretation by human experts (clinical scientists with experience of reading LFIAs). The samples were selected randomly or were selected as they fulfilled a certain criteria (Sample was submitted by a participant, who was vaccinated 21+ days prior, and reported a negative result).

Blinding

Human experts were blinded to participant interpretation of test results when asked to interpret the test results independently for determining concordance of test result interpretation between experts and study participants.

Reporting for specific materials, systems and methods

We require information from authors about some types of materials, experimental systems and methods used in many studies. Here, indicate whether each material, system or method listed is relevant to your study. If you are not sure if a list item applies to your research, read the appropriate section before selecting a response.

Materials & experimental systems

n/a	Involved in the study	
☒	☐	Antibodies
☒	☐	Eukaryotic cell lines
☒	☐	Palaeontology and archaeology
☒	☐	Animals and other organisms
☐	☒	Human research participants
☒	☐	Clinical data
☒	☐	Dual use research of concern

Methods

n/a	Involved in the study	
☒	☐	ChIP-seq
☒	☐	Flow cytometry
☒	☐	MRI-based neuroimaging

Human research participants

Policy information about studies involving human research participants

Population characteristics	Co-variate relevant population characteristics are not applicable to the study described in this manuscript. However, characteristics of REACT-2 study participants, from which the images were obtained, can be found in published manuscripts describing the sero-prevalence results:<br>Ward, H. et al. Prevalence of antibody positivity to SARS-CoV-2 following the first peak of infection in england: serial cross-sectional studies of 365,000 adults. The Lancet Regional Health Europe 4 (2021). https://www.thelancet.com/journals/lanepe/article/PIIS2666-7762(21)00075-2/fulltext<br><br>Ward, H., Atchison, C., Whitaker, M. et al. SARS-CoV-2 antibody prevalence in England following the first peak of the pandemic. Nat Commun 12, 905 (2021). https://doi.org/10.1038/s41467-021-21237-w
Recruitment	For each round of our survey, we contacted a random sample of the population by sending a letter to named individuals aged 18 or over from the National Health Service (NHS) patient list (almost the whole population).
Ethics oversight	South Central-Berkshire B Research Ethics Committee (IRAS ID: 283787)

Note that full information on the approval of the study protocol must also be provided in the manuscript.
